# Gastric cancer cells-derived exosomal miR-151a-5p induces an immunosuppressive microenvironment through promoting LAG3^+^TAMs infiltration

**DOI:** 10.1186/s13046-026-03703-9

**Published:** 2026-04-01

**Authors:** Peng Zhou, Huiheng Qu, Yu Tang, Kaihang Shi, Zequn Zhuang, Chen Qiu, Yupeng Zhao, Youwei Han, Zhihui Yang, Yuyan Ding, Tianlu Jiang, Shuai Liang, Kaiyuan Deng, Yigang Chen, Jiazeng Xia

**Affiliations:** 1 Department of General Surgery / Research Institute of General Surgery, Wuxi No.2 People’s Hospital, Jiangnan University Medical Center, Wuxi, Jiangsu 214000 China; 2 Department of General Surgery, The Affiliated Wuxi No.2 People’s Hospital, Wuxi Medical Center of Nanjing Medical University, Wuxi, Jiangsu China; 3 Department of Hepatobiliary Surgery, The Affiliated Yixing Hospital of Jiangsu University, Wuxi, Jiangsu China; 4 Department of Hepatobiliary Surgery, Jinjiang Municipal Hospital (Shanghai Sixth People’s Hospital Fujian), Quanzhou, Fujian China; 5 Department of General Surgery, Zhongda Hospital Southeast University, Nanjing, Jiangsu China; 6 Department of Breast Surgery/General Surgery, The Affiliated Wuxi People’s Hospital, Wuxi Medical Center of Nanjing Medical University, Wuxi, Jiangsu China; 7 Department of Radiation Oncology, Weifang People’s Hospital, Shandong Second Medical University, Weifang, China

**Keywords:** Gastric cancer, Tumor-associated macrophage, Immunotherapy, Lymphocyte-activation gene 3, Exosome

## Abstract

**Background:**

The incidence and mortality of gastric cancer (GC) are still in the forefront worldwide. Up to now, the population benefiting from immunotherapy is still very limited. Tumor associated macrophages (TAM) play an important role in immune response and immune microenvironment remodeling. This study aimed to clarify the altered TAM subsets in GC microenvironment and elucidate the internal molecular mechanism.

**Methods:**

Sample collection and Cytometry by Time-of-Flight (CyTOF) were used to identify TAM subpopulations with differential infiltration in the GC microenvironment, followed by multiplex immunofluorescence and flow cytometry to validate the immunosuppressive function. Exosomes were isolated and characterized by transmission electron microscopy, nanoparticle tracking analysis and western blot for subsequent high-throughput RNA sequencing. The key regulatory role of LAG3^+^ TAMs in the progression of gastric cancer was explored by Chromatin Immunoprecipitation, dual luciferase reporter, cytokine array and subcutaneous xenograft models.

**Results:**

The infiltration of LAG3^+^ TAMs was elevated in GC and correlated with poorer prognosis. LAG3^+^ TAMs significantly suppressed the effector function and cytotoxic activity of CD8^+^ T cells. Mechanistically, GC cells-derived exosomes delivered miR-151a-5p to macrophages, inducing LAG3 expression via DUSP8/MAPK-dependent activation of ELK1 transcription. Reciprocally, LAG3^+^ TAMs released CXCL8, which bound CXCR2 on GC cells, amplifying exosomal miR-151a-5p secretion and reinforcing the immunosuppressive loop.

**Conclusions:**

LAG3⁺ TAMs are expanded in GC through a tumor-macrophage feedback circuit driven by exosomal miR-151a-5p, resulting in CD8⁺ T-cell dysfunction and immune resistance. Targeting this axis may enhance GC immunotherapy efficacy.

**Graphical Abstract:**

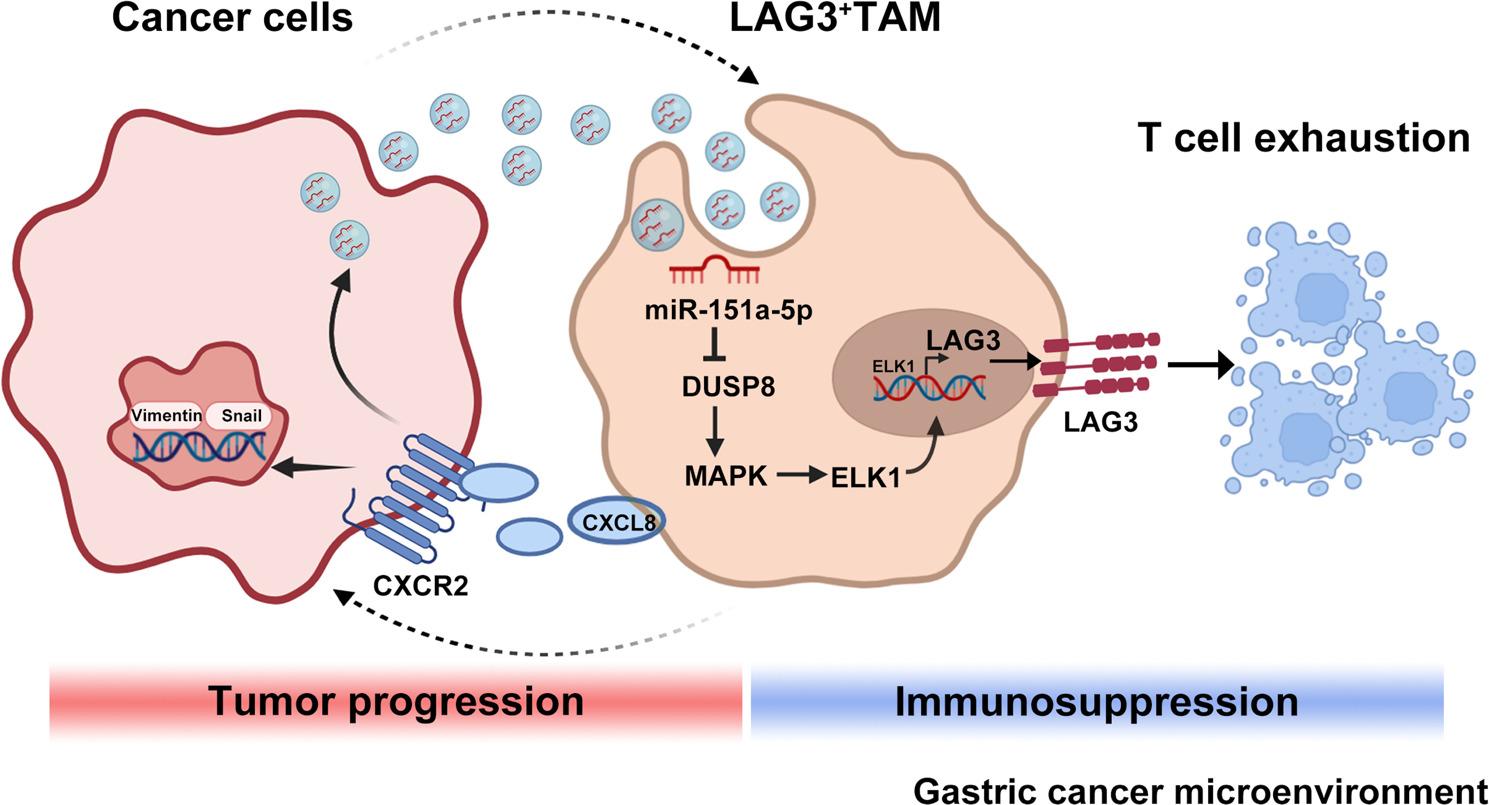

**Supplementary Information:**

The online version contains supplementary material available at 10.1186/s13046-026-03703-9.

## Background

According to the latest global cancer statistics, there were over 20 million new cancer cases worldwide in 2022, with GC accounting for 4.9% of incident cases and 6.8% of cancer-related deaths, both ranking fifth [1]. Although advances in endoscopic technology have significantly improved the detection rate of early-stage GC, approximately 80–90% of patients are still diagnosed at advanced stages. The conventional treatment strategy, D2 radical gastrectomy combined with chemotherapy, offers limited efficacy, with a median overall survival typically under one year [2]. In recent years, immunotherapy, particularly immune checkpoint inhibitors (ICIs), has emerged as a transformative approach by reversing immune suppression and restoring anti-tumor immune responses [3]. Based on data from the phase III KEYNOTE-859 trial, the addition of pembrolizumab to chemotherapy significantly prolonged median overall survival by 1.4 months compared to chemotherapy alone in patients with HER2-negative GC, reducing the risk of death by 22% [4]. This combination regimen has since been incorporated into international clinical practice guidelines [5]. Nevertheless, the clinical benefits of immunotherapy in GC remain constrained by several challenges, including low objective response rates, complex mechanisms of acquired resistance, immune-related adverse events (irAEs) and insufficient biomarkers for predicting treatment response [6]. Tumor immune microenvironment (TiME) heterogeneity represents a key factor underlying the variable efficacy and limitations of immunotherapy in GC [7].

The TiME comprises tumor cells, immune cells, stromal cells and the vascular system, forming a complex ecosystem that surrounds and infiltrates tumor tissues. TiME plays a crucial role in regulating tumor progression, metastasis and responses to treatment. The composition, functional status and molecular signaling of immune cells within the same type of tumor can vary significantly among different patients. Understanding the TiME of GC is particularly important for developing personalized treatment strategies and improving therapeutic outcomes. Macrophages originate from bone marrow hematopoietic stem cells (HSCs) and gradually differentiate into myeloid and monocyte lineages after entering the bloodstream. Under the influence of macrophage colony-stimulating factor (M-CSF) and other stimuli, they ultimately differentiate into macrophages [8]. As key components of the innate immune system, macrophages play central roles in immune regulation, pathogen phagocytosis, antigen presentation, and modulation of tissue repair and inflammatory responses [9]. However, in the TiME, tumor-associated macrophages (TAMs), one of the most abundant immune cell types, have significant impacts on tumor progression and immune regulation. Recently, a study published in *Nature* reported that blocking the adenosine A2A receptor (A2AR) in advanced prostate cancer not only effectively reduced the immunosuppressive effects of SPP1hi-TAMs but also significantly enhanced the efficacy of ICIs [10]. Despite extensive evidence showing that TAMs provide tumor growth factors, promote angiogenesis, and enhance tumor cell invasion and metastasis, their prognostic value remains controversial, likely due to its high heterogeneity. For example, FOLR2 + macrophages are associated with better prognosis in breast cancer patients, whereas C1q+ macrophages secrete Ebi3 to induce T-cell exhaustion and promote tumor progression. Therefore, a deeper understanding of TAM heterogeneity is essential for advancing the immune microenvironment. Immunotherapy based on macrophages has become a research hotspot in cancer treatment. It aims to reprogram TAMs in situ [11] and utilize CAR-macrophage therapy [12] to modulate macrophage polarization states [13], as well as interactions between macrophages and other immune cells [14], thereby reshaping the immunosuppressive microenvironment and enhancing the sensitivity to immunotherapy.

Exosomes are nanosized extracellular vesicles (30–150 nm in diameter) secreted by cells and enriched with biomacromolecules such as proteins, lipids and nucleic acids [15]. They play critical roles in intercellular communication, material transfer and signal transduction by delivering bioactive molecules to recipient cells, thereby modulating cellular functions [16, 17]. For cancer immunotherapy, exosomes have emerged as potential carriers for inducing anti-tumor immune responses and enabling targeted delivery of therapeutic agents to specific cell types, thus regulating cellular phenotypes and immunomodulatory capacities [18–20]. For instance, Liang et al. reported that highly metastatic colorectal cancer cells secrete exosomal miR-106a-5p, which induced macrophage polarization toward the M2 phenotype in the TME, promoting liver metastasis and correlating with poor prognosis [21]. Similarly, the Huang’s group demonstrated that colorectal cancer cell-derived exosomal miR-21-5p and miR-200 acted synergistically to drive M2-like polarization of macrophages and upregulate PD-L1 expression, leading to increased infiltration of PD-L1^+^CD206^+^ macrophages. This may indicate a potential strategy to sensitize colorectal cancer to anti-PD-L1 immunotherapy [22]. Exosomes mediate intercellular communication by delivering bioactive molecules, including circRNAs, miRNAs, and small proteins, thereby promoting immunosuppressive macrophage polarization and fostering an immune-evasive tumor microenvironment.

In this study, we employed mass cytometry (CyTOF) integrated with protein expression profiling and cell type annotation to characterize the cellular subpopulations and their phenotypic heterogeneity within the GC microenvironment. We identified a tumor-infiltrating TAM subset that significantly enriched in GC, LAG3⁺ TAMs. Through a combination of in vitro cellular assays, patient cohort analysis and animal models, we elucidated the role of LAG3⁺ TAMs in promoting immune evasion in gastric cancer and uncovered the underlying molecular mechanisms. These findings may provide novel insights into enhancing the sensitivity of GC patients to immunotherapy.

## Methods

### Patient specimens

Fresh-frozen GC and matched adjacent non-tumor tissues used for CyTOF, Multiplex immunohistochemistry(mIHC) and flow cytometry were obtained from GC patients undergoing surgery resection at the Department of General Surgery, Wuxi No.2 People’s Hospital. Immediately after surgical resection and tissue dissociation, samples were snap-frozen in liquid nitrogen and stored for subsequent analysis. All tissue specimens were histopathologically confirmed by two independent pathologists according to the criteria of the 8th AJCC edition. None of the patients had received preoperative chemotherapy, radiotherapy, or immunotherapy prior to surgery. This study was approved by Ethics Committee of the Wuxi No.2 People’s Hospital (Approval No. Y-132), and written informed consent was obtained from all participants.

Other experimental methods used are detailed in Supplementary Material 2 and Table 1.

**Table 1:** Primer sequence

	Sequence
LAG3	Forward: ACAGGTGAGCCAGGGACAT
	Reverse: TCTTGGTCGCCACTTCCTGA
ELK1	Forward: ACTCCTCCGCATCCCTCTTT
	Reverse: TCCCGTGAAGTCCAGGAGAT
CBX7	Forward: GAGACCGAGCATCGGGGTAT
	Reverse: CAGAGCTTCTCCTTGCCCTTG
DUSP8	Forward: CTGCCAAGTCATCGTCCACT
	Reverse: GGACATGCCCATGGTCTTCA
NPR2	Forward: AGCTGATGCTGGAGAAGGAG
	Reverse: ACTGCCTGCACCTTTGTGAT
PPP1R12B	Forward: AGTGAGGTGGCCAATTCCAG
	Reverse: ATTCCCCCATACCCTCCCAA
SVEP1	Forward: GCTCCCATCTCTCCATGCTC
	Reverse: TGTCGCTGCCGTTATCAACT
miR-151a-5p	UUGUACUACACAAAAGUACUG
miR-151a-5p mimics	UCGAGGAGCUCACAGUCUAGU
miR-151a-5p inhibitor	ACUAGACUGUGAGCUCCUCGA

## Results

### 3.1. LAG3^+^ TAMs was identified in GC microenvironment

**Fig. 1:**
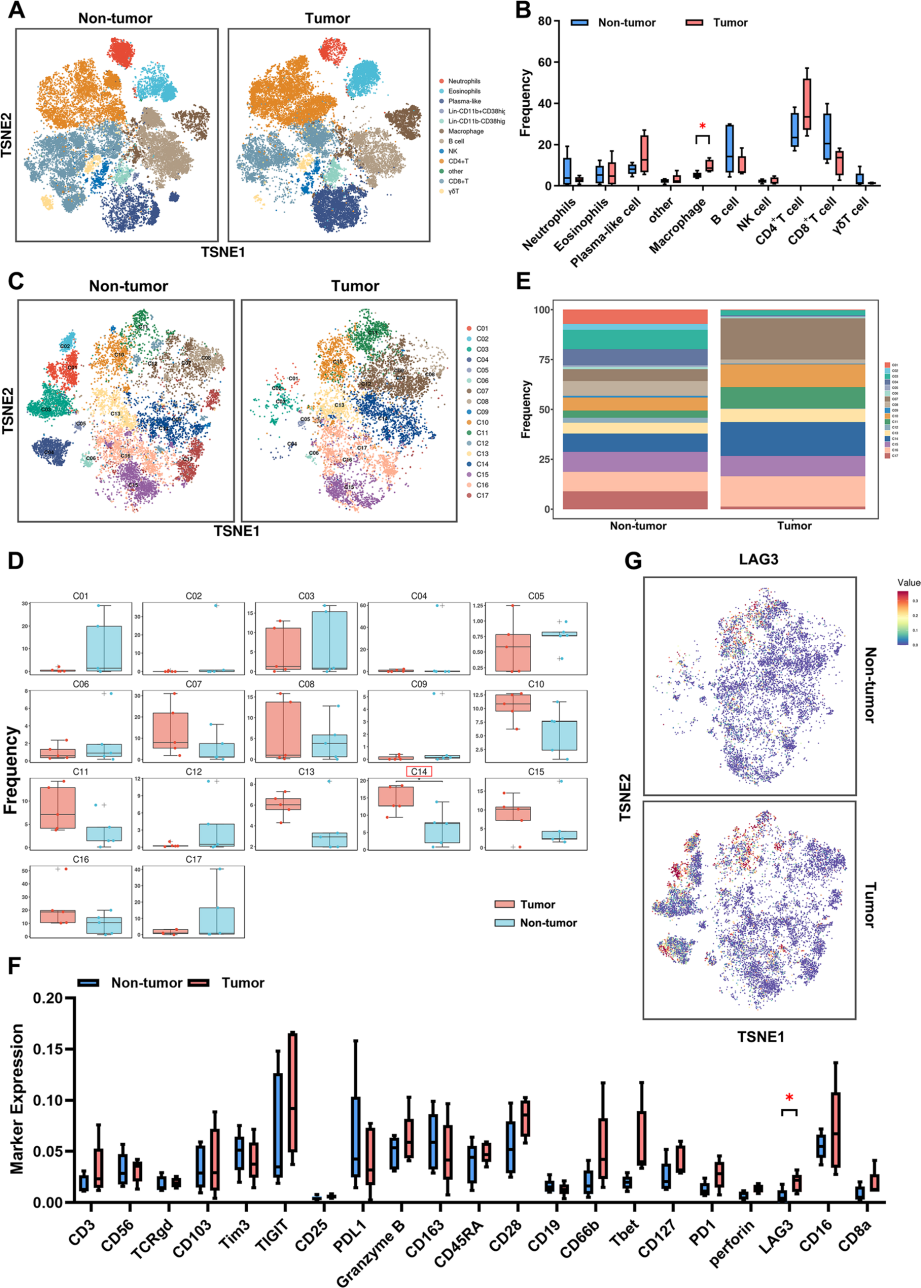
LAG3^+^TAMs subset was identified in GC microenvironment. **A** Immune cell infiltration profiling in GC tissues. **B** Frequency of immune cell infiltration in GC tissues and paired non-tumor tissues. **C** Differential infiltration of macrophage subtypes in GC tissues and paired non-tumor tissues. **D-E** Frequency of differential infiltration of macrophage subtypes. **F** Cell surface marker expression in C14 macrophage subpopulations (part 2). **G** Expression of LAG3 in C14 macrophage subsets in GC tissues and paired non-tumor tissues. *p*-values were determined by two-tailed unpaired Student’s *t*-test. *p < 0.05 versus the control group

To investigate the immune landscape of GC and identify specific immune cell subtypes with potential functional significance, we collected paired GC and non-tumor tissues for CyTOF. Unsupervised clustering identified 12 major immune cell populations enriched in GC microenvironment (Fig. S1A-B). To visualize the spatial distribution of these subsets, we performed t-distributed Stochastic Neighbor Embedding (t-SNE) dimensionality reduction on the CyTOF data. This revealed distribution patterns of immune cells, with marked difference in macrophage infiltration (Fig. 1A-B). Macrophages were then reclustered into 17 distinct clusters based on differential expression of surface markers (Fig. S1C-D). Abundance analysis and phenotypic profiles identified C14 subset as significantly enriched in tumor tissues compared to non-tumor tissues (Fig. 1C-E). Based on analysis of marker expression within the C14 cluster, we identified a TAM subpopulation characterized by high LAG3 expression (Fig. 1F-G and Fig. S1E); accordingly, we designated this cluster as LAG3^+^TAMs in the present study.

### Elevated LAG3^+^ TAMs in GC were correlated with CD8^+^ T cell exhaustion

Lymphocyte Activation Gene 3 (LAG3), also known as CD223, is a member of the immunoglobulin superfamily (IgSF) and shares structural homology with CD4, as well as overlapping functional properties [23]. LAG3 is predominantly expressed on activated T cells and natural killer (NK) cells in humans, where it acts synergistically with PD-1 to negatively regulate T cell proliferation, activation and homeostasis, thereby contributing to tumor immune evasion [24, 25]. Preclinical studies have demonstrated that co-expression of LAG-3 and PD-1 on CD8^+^ T cells leads to synergistic inhibition, resulting in T cell exhaustion and impaired autocrine IFN-γ-dependent antitumor immune responses [26]. Clinically, the combination of relatlimab (an anti-LAG-3 monoclonal antibody) and nivolumab (an anti-PD-1 antibody) has been shown to enhance CD8^+^ T cells receptor signaling and promote favorable differentiation of CD8^+^ T cells, culminating in increased cytotoxic activity. Treatment with this dual checkpoint blockade regimen induces a marked expansion of CD8^+^ T cells with enhanced effector phenotypes, as evidenced by an increased frequency of CD38^+^TIM3^+^CD8^+^ T cells in peripheral blood-a change that correlates with improved clinical outcomes (NCT03743766) [27]. These findings underscore the therapeutic potential of LAG-3 inhibition and support its integration into next-generation cancer immunotherapies.

**Fig. 2:**
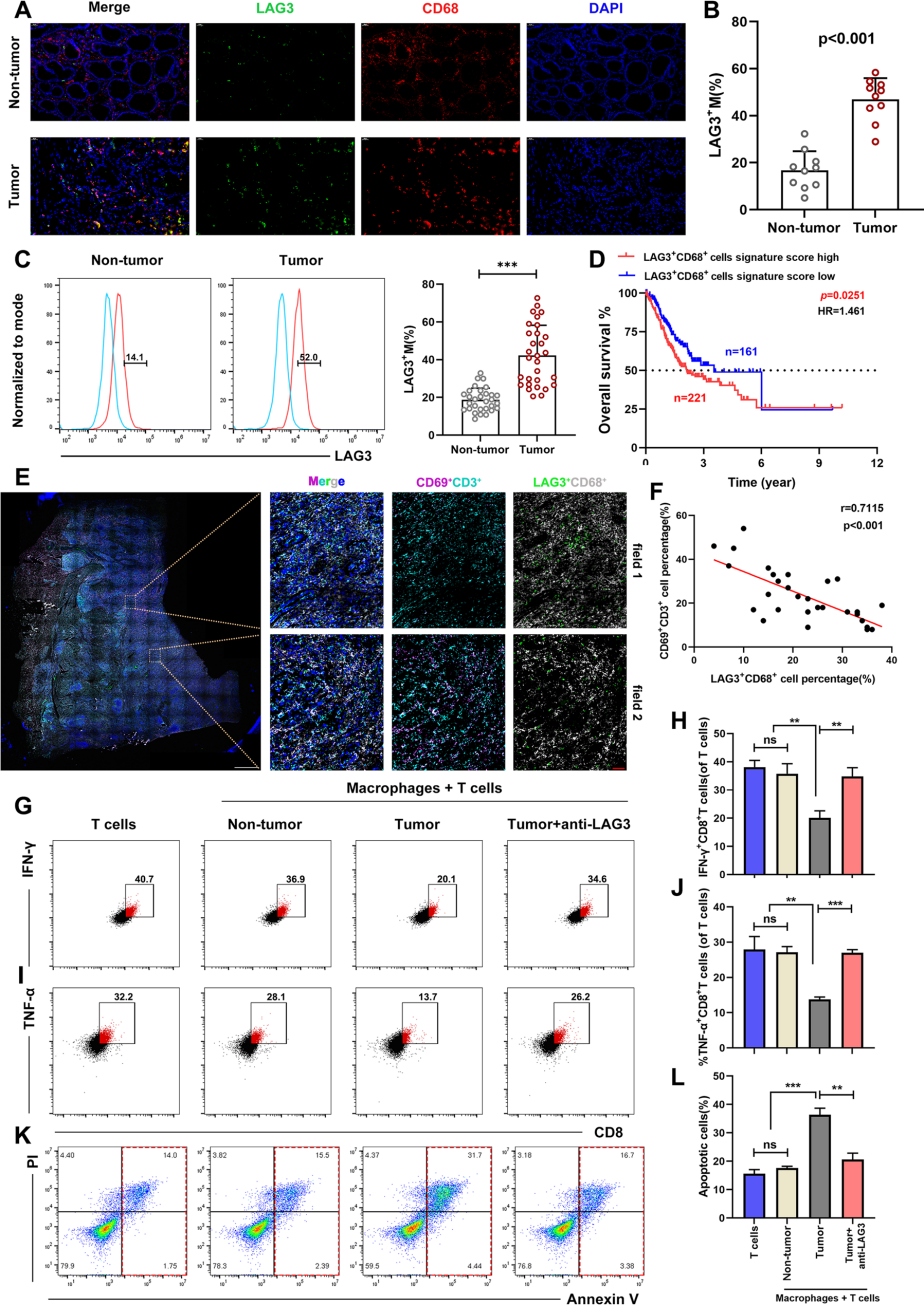
Elevated LAG3^+^TAMs in GC were correlated with CD8^+^T cell exhaustion. **A-B** mIHC staining for LAG3 and CD68 in GC tissues and paired non-tumor tissues (*n* = 10). **C** Comparative flow cytometry analysis of LAG3 expression in macrophages from GC and paired non-tumor tissues (*n* = 30). **D** Survival analysis of GC patients stratified by LAG3^+^CD68^+^ TAM infiltration levels in the TCGA cohort. **E-F** mIHC imaging of CD69^+^CD3^+^T cells and LAG3^+^TAMs in GC tissues (*n* = 30). **G-H** IFN-γ production of T cells after co-culture with LAG3^+^TAMs ± LAG3-neutralizing antibodies. **I-J** TNF-α production of T cells after co-culture with LAG3^+^TAMs ± LAG3-neutralizing antibodiess. **K-L** Apoptosis levels of T cells after co-culture with LAG3^+^TAMs ± LAG3-neutralizing antibodies. All experiments were repeated 3 times with consistent results. The data are presented as the means ± SD. *p*-values were determined by two-tailed unpaired Student’s *t*-test. **p < 0.01, ***p < 0.001, “ns” not significant versus the control group

To further validate the infiltration level of LAG3^+^TAMs in GC, we first conducted multiplex immunohistochemistry (mIHC) of ten paired GC and non-tumor tissues, which revealed a significantly higher abundance of LAG3^+^TAMs in tumor tissues compared to non-tumor counterparts (Fig. 2A-B). Flow cytometry analysis of an expanded cohort corroborated these findings, demonstrating a marked increase in LAG3^+^TAMs within the GC microenvironment that aligned with the mRNA expression data from the same cohort (Fig. 2C and Fig. S2A).

Based on LAG3^+^TAM signature, the analysis of the TCGA stomach adenocarcinoma cohort revealed that patients with high signature scores had significantly worse overall survival compared to those with low scores (log-rank *p* = 0.0251, HR = 1.461; Fig. 2D). Furthermore, the high-score group exhibited significantly elevated Z-scores for immune checkpoint genes including TIGIT, CTLA4 and PDCD1, suggesting a strong association between LAG3^+^TAMs and an immunosuppressive microenvironment (Fig. S2B).

Given the well-documented role of TAMs in suppressing T cell function [28, 29], we next investigated the potential interaction between LAG3^+^TAMs and T cells. mIHC revealed that regions with high infiltration of LAG3^+^TAMs exhibited reduced activated (CD69^+^) T cells, whereas areas with lower LAG3^+^TAMs density showed greater enrichment of activated T cells (Fig. 2E-F), indicating a negative spatial correlation between LAG3^+^ TAMs and T cell activation. To evaluate the functional impact of LAG3^+^TAMs on T cell immune responses, LAG3^+^TAMs were isolated from paired GC and non-tumor tissues and then co-cultured with autologous peripheral blood CD3^+^T cells. Interferon-γ (IFN-γ) and Tumor Necrosis Factor α (TNF-α) are critical effector cytokines produced by cytotoxic CD8^+^ T cells during anti-tumor immune responses [30, 31]. Notably, compared to those co-cultured with non-tumor-derived macrophages, T cells co-cultured with tumor-derived macrophages exhibited significantly reduced IFN-γ and TNF-α (Fig. 2G-J). In addition, T cells exposed to tumor-derived LAG3^+^TAMs displayed higher apoptosis rates (Fig. 2K-L). Strikingly, the immunosuppressive effects of tumor-derived macrophages on T cell cytokine production and survival were significantly reversed upon treatment with LAG3-neutralizing antibodies (Fig. 2G-L). Meanwhile, the functional assays demonstrated that while LAG3 antibodies alone exert a modest effect on T cell activation, the promoting effect on T cell effector function was significantly enhanced in the presence of macrophages compared to the effects on T cells alone (Fig. S2C-E). Taken together, the results suggested that the LAG3 pathway plays a functional role in mediating TAM-induced T cell exhaustion.

### GC cells-derived exosomes promoted LAG3^+^TAM infiltration and mediated T cell suppression

Exosomes are well-established mediators of intercellular communication, capable of transferring bioactive molecules that modulate immune responses within the tumor microenvironment [32]. To investigate whether exosomes contribute to the LAG3^+^TAMs-mediated exhaustion of T cells in GC, we isolated exosomes from the supernatants of the immortalized gastric epithelial cell line GES-1 and GC cells (AGS, HGC27, and MKN45) using differential ultracentrifugation, followed by comprehensive identification and functional characterization.

**Fig. 3:**
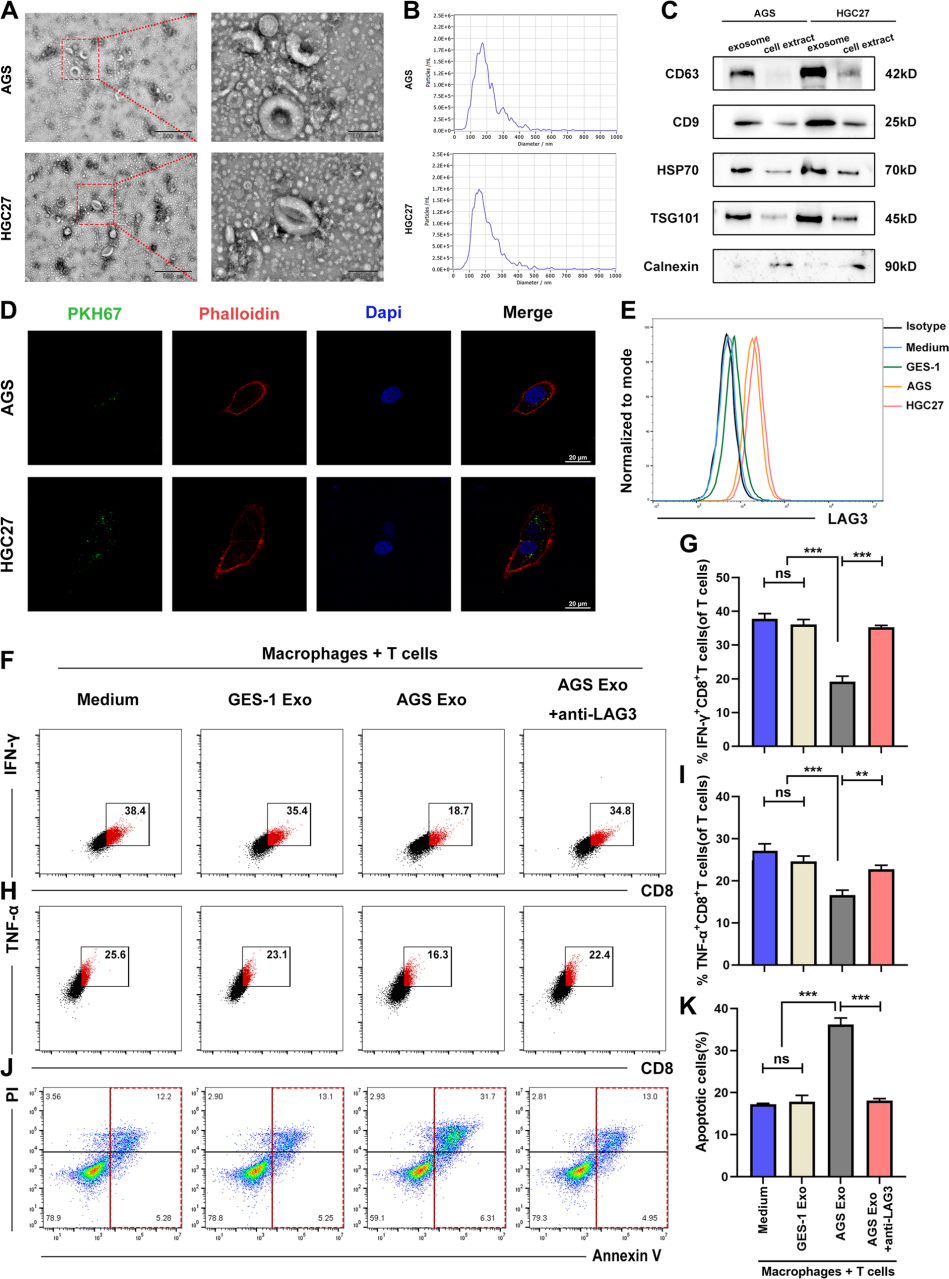
GC cells-derived exosomes promoted LAG3^+^TAM differentiation and mediated T cell suppression. **A** TEM image of GC cells-derived exosomes showing cup-shaped morphology. **B** Mean diameter of exosomes from GC cells by NTA. **C** Exosomal markers (CD63, CD9, TSG101, HSP70) and Calnexin (negative control) in GC cells-derived exosomes. **D** PKH67-labeled exosome (green) uptake by phalloidin-labeled TAMs (red). **E** Flow cytometry analysis of LAG3 expression on macrophages following treatment with GC cells-derived exosomes. **F-G** IFN-γ production by T cells in co-culture with AGS-derived-exosome treated LAG3^+^ TAMs ± LAG3-neutralizing antibodies. **H-I** TNF-α production by T cells in co-culture with AGS-derived-exosome treated LAG3^+^ TAMs ± LAG3-neutralizing antibodies. **J-K** Apoptosis levels of T cells in co-culture with AGS-derived-exosome treated LAG3^+^ TAMs ± LAG3-neutralizing antibodies. All experiments were repeated 3 times with consistent results. The data are presented as the means ± SD. *p*-values were determined by two-tailed unpaired Student’s *t*-test. **p < 0.01, ***p < 0.001, “ns” not significant versus the control group

Transmission electron microscopy (TEM) revealed that the isolated exosomes exhibited characteristic cup-shaped morphology (Fig. 3A). Nanoparticle Tracking Analysis (NTA) confirmed a size distribution predominantly between 50 and 200 nm, consistent with typical exosomal dimensions (Fig. 3B). Western blot analysis further validated the presence of canonical biomarkers of exosomes (CD63, CD9, HSP70 and TSG10), while the endoplasmic reticulum-resident protein Calnexin was undetectable, confirming minimal contamination from cellular organelles (Fig. 3C).

To assess the uptake of exosomes by macrophages, we labeled GC cells-derived exosomes with PKH67 and co-cultured them with macrophages stained with phalloidin. Confocal microscopy showed the internalization of PKH67-labeled exosomes by macrophages (Fig. 3D), indicating efficient exosome-macrophage interaction. Given that, we next examined whether GC cells-derived exosomes could modulate macrophage polarization toward the LAG3^+^TAM phenotype. Flow cytometry revealed that macrophages co-cultured with exosomes from AGS and HGC27 exhibited a significantly higher proportion of compared to those treated with GES-1–derived exosomes (Fig. 3E and Fig. S3A), suggesting that tumor-derived exosomes promote the infiltration of this LAG3^+^ TAMs subset.

We then evaluated the functional consequences of exosome-induced LAG3^+^ TAMs on CD8^+^ T cell activity. Macrophages pre-treated with GC cells-derived exosomes were co-cultured with T cells and flow cytometry demonstrated that T cells co-cultured with AGS exosome–primed macrophages exhibited markedly reduced production of IFN-γ and TNF-α (Fig. 3F–I), compared to those co-cultured with GES-1-derived exosome or untreated macrophages. Moreover, these macrophages significantly enhanced the apoptosis of T cells (Fig. 3J–K). Notably, the immunosuppressive effects of GC exosome–educated macrophages on T cell cytokine production and survival were substantially reversed upon treatment with LAG3-neutralizing antibodies (Fig. 3F–K), Similar results were observed with HGC27-derived exosomes, which also induced LAG3^+^ TAM infiltration and subsequent T cell suppression (Fig. S3B–G), indicating that the LAG3 pathway is functionally involved in this exosome-driven immunosuppressive axis.

### GC cells-derived exosomal miR-151a-5p was transferred to macrophages and promoted LAG3 expression

**Fig. 4:**
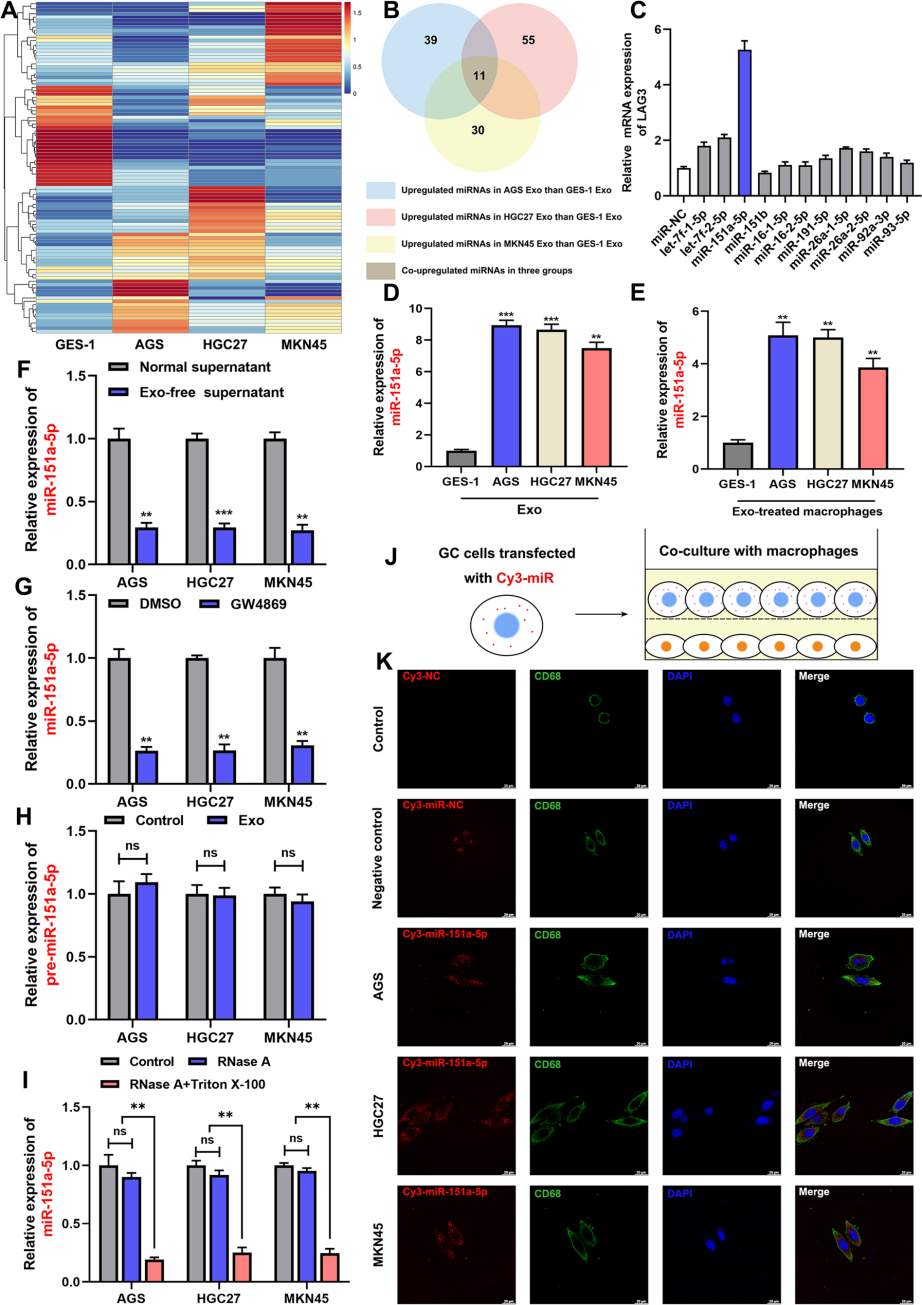
GC cells-derived exosomal miR-151a-5p was transferred to macrophages and promoted LAG3 expression. **A** Heatmap of transcriptomic profiling in exosomes from normal gastric epithelial cells (GES-1) and GC cell lines (AGS, HGC27, MKN45). **B** Venn diagram analysis of overlapping and unique miRNAs in exosomes derived from GC cell lines. **C** qRT-PCR analysis of the regulatory effects of candidate miRNA mimics on LAG3 expression in macrophages. **D** Relative expression of miR-151a-5p in exosomes from GES-1, AGS, HGC27, and MKN45. **E** qRT-PCR analysis of miR-151a-5p levels in LAG3^+^ TAMs after co-culture with GC cells-derived exosomes. **F** Levels of miR-151a-5p in LAG3^+^TAMs after co-culture with exosome-depleted supernatant from GC cells. **G** Levels of miR-151a-5p in LAG3^+^TAMs after co-culture with supernatant from GC cells treated with GW4869. **H** Relative expression of pre-miR-151a-5p in LAG3^+^ TAMs after co-culture with GC cells-derived exosomes. **I** Levels of miR-151a-5p in LAG3^+^ TAMs after treatment of RNase A and/or Triton X-100. **J-K** Cy3 fluorescence tracing of miR-151a-5p transfer from transfected GC cells to LAG3^+^ TAMs after co-culture. All experiments were repeated 3 times with consistent results. The data are presented as the means ± SD. *p*-values were determined by two-tailed unpaired Student’s *t*-test. **p < 0.01, ***p < 0.001, “ns” not significant versus the control group

Exosomal miRNAs serve as key mediators of intercellular communication among immune cells, orchestrating their metabolic reprogramming, modulating their gene expression profiles, and contributing to the maintenance of immune microenvironment homeostasis. To identify whether exosomal miRNAs mediate the reprogram of macrophages toward LAG3^+^ TAM phenotype in GC, we performed transcriptomic sequencing on exosomes isolated from GC cell lines and GES-1 cells (Fig. 4A). A Venn diagram analysis identified 11 miRNAs consistently highly expressed in GC cells-derived exosomes but not in GES-1–derived exosomes (Fig. 4B). To determine which of these miRNAs could regulate LAG3 expression in macrophages, we transfected macrophages with miRNA mimics corresponding to the 11 candidates and assessed *LAG3* mRNA levels by qRT-PCR. Among them, miR-151a-5p induced the most pronounced upregulation of LAG3, suggesting its potential role in promoting the LAG3^+^ TAM phenotype (Fig. 4C).

We next confirmed that miR-151a-5p was significantly enriched in exosomes derived from GC cell lines and GC patients (Fig. 4D and Fig. S4A–B). Moreover, macrophages treated with GC cells-derived exosomes exhibited significantly higher levels of miR-151a-5p than those treated with GES-1–derived exosomes (Fig. 4E). To confirm that miR-151a-5p delivery was exosome-dependent, we generated exosome-depleted supernatants by high-speed centrifugation or inhibited exosome biogenesis using GW4869, an inhibitor of neutral sphingomyelinase. Macrophages cultured with either exosome-depleted supernatants or in the presence of GW4869 showed significantly reduced miR-151a-5p levels compared to those exposed to normal supernatants (Fig. 4F–G), supporting the necessity of exosomes for efficient transfer. Notably, no significant change in the expression of pre-miR-151a-5p was observed in macrophages after exosome treatment (Fig. 4H), suggesting that the increased miR-151a-5p levels were due to exogenous transfer of the mature miRNA rather than de novo transcription and processing within macrophage. We then treated GC cell supernatants with RNase A alone or in combination with the membrane-permeabilizing agent Triton X-100. RNase A alone did not reduce miR-151a-5p levels in recipient macrophages, whereas the addition of Triton X-100 led to a significant decrease (Fig. 4I), confirming that miR-151a-5p was protected within lipid-bilayer vesicles. Finally, to directly visualize intercellular transfer, we transfected GC cells with Cy3-labeled miR-151a-5p and co-cultured them with macrophages for 24 h (Fig. 4J). Fluorescence microscopy revealed clear Cy3 (red) signal within macrophages (Fig. 4K), providing direct evidence of miR-151a-5p transfer from GC cells to macrophages.

Collectively, these findings demonstrated that miR-151a-5p was selectively packaged into GC cells-derived exosomes, transported to macrophages and contributed to the immunosuppressive LAG3^+^ TAM.

### GC cells-derived exosomal miR-151a-5p prompted LAG3^+^TAM infiltration and impaired T cell function

To determine whether exosomal miR-151a-5p contributes to immune evasion by promoting the infiltration of LAG3^+^TAMs, we functionally modulated miR-151a-5p levels in GC cells and assessed its subsequent immunomodulatory effects.

**Fig. 5:**
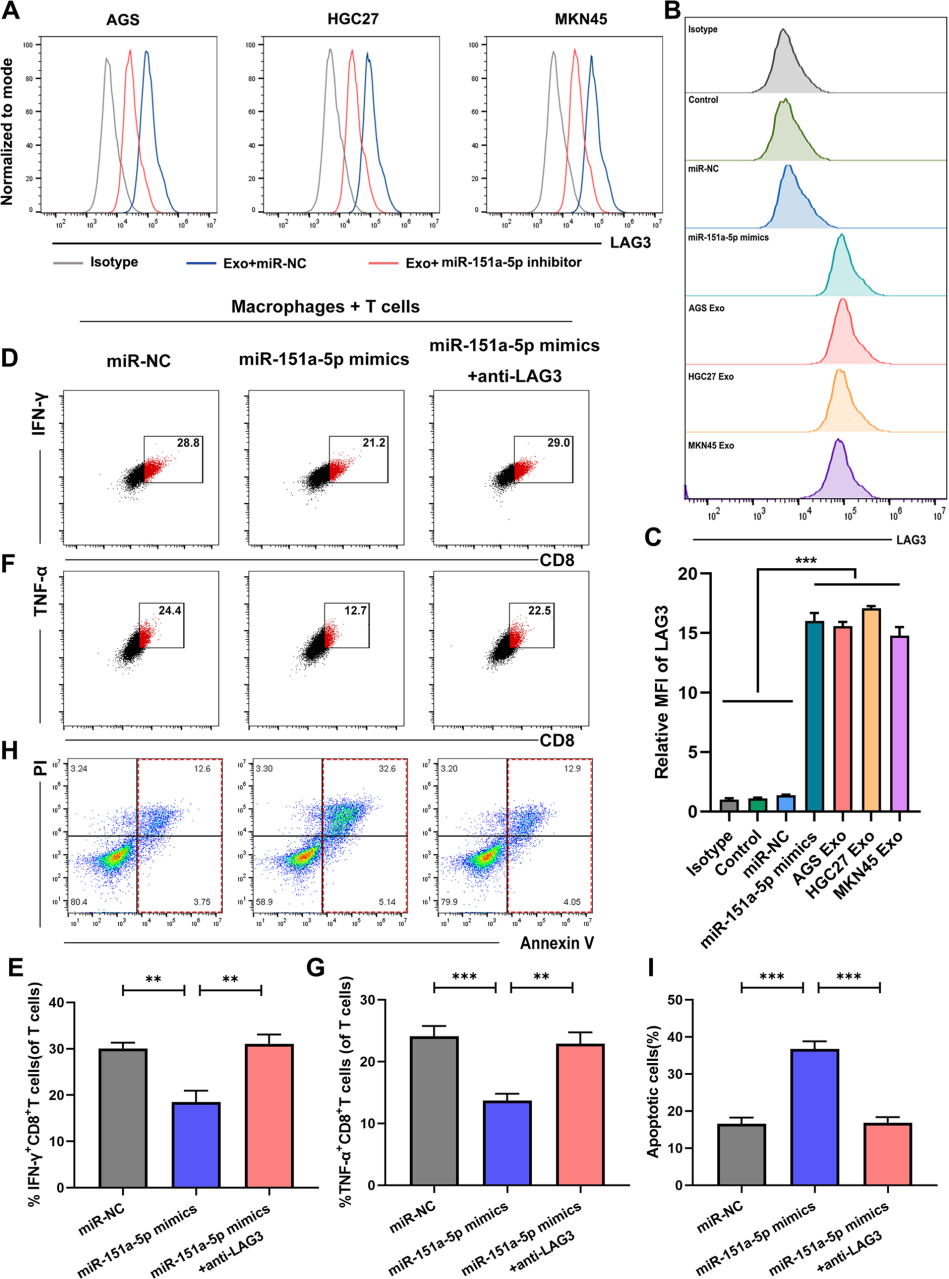
GC-derived exosomal miR-151a-5p prompted LAG3^+^ TAM infiltration and impaired T cell function. **A** Flow cytometry analysis of LAG3 expression after co-culture with exosomes derived from GC cells transfected with miR-NC or miR-151a-5p inhibitor. **B-C** The levels of LAG3 in LAG3^+^TAMs measured by flow cytometry following either direct transfection with miR-151a-5p mimics or co-incubation with GC cells-derived exosomes. **D-E** IFN-γ production by T cells co-cultured with miR-151a-5p mimic-transfected LAG3^+^TAMs ± LAG3-neutralizing antibodies. **F-G** TNF-α production by T cells co-cultured with miR-151a-5p mimic-transfected LAG3^+^TAMs ± LAG3-neutralizing antibodies. **H-1** Apoptosis levels of T cells co-cultured with miR-151a-5p mimic-transfected LAG3^+^TAMs ± LAG3-neutralizing antibodies. All experiments were repeated 3 times with consistent results. The data are presented as the means ± SD. *p*-values were determined by two-tailed unpaired Student’s *t*-test. **p < 0.01, ***p < 0.001 versus the control group

First, we designed and validated a specific miR-151a-5p inhibitor in AGS, HGC27 and MKN45 cells (Fig. S5A). Flow cytometry and qRT-PCR analysis revealed that macrophages exposed to miR-151a-5p–deficient exosomes exhibited significantly reduced LAG3 expression compared to those treated with control exosomes (Fig. 5A and Fig. S5B). Conversely, transfection of macrophages with miR-151a-5p mimics resulted in a marked upregulation of LAG3, similar to the macrophages treated with GC cells-derived exosomes (Fig. 5B-C).

Macrophages transfected with miR-151a-5p mimics and subsequently co-cultured with T cells led to a significant reduction in IFN-γ and TNF-αproduction (Fig. 5D-G), along with increased T cell apoptosis (Fig. 5H-I). To identify the functional role of LAG3 in this immunosuppressive phenotype, we introduced a LAG3-neutralizing antibody into the co-culture system and effectively reversed the suppressive effects on T cell function (Fig. 5D-I), confirming the involvement of LAG3 in mediating immune inhibition. Additionally, treatment with the miR-151a-5p inhibitor attenuated the immunosuppressive properties of GC cells-derived exosomes on macrophages (Fig. S6A–F).

### GC cells-derived exosomal miR-151a-5p regulated DUSP8/MAPK signaling axis in macrophages

**Fig. 6:**
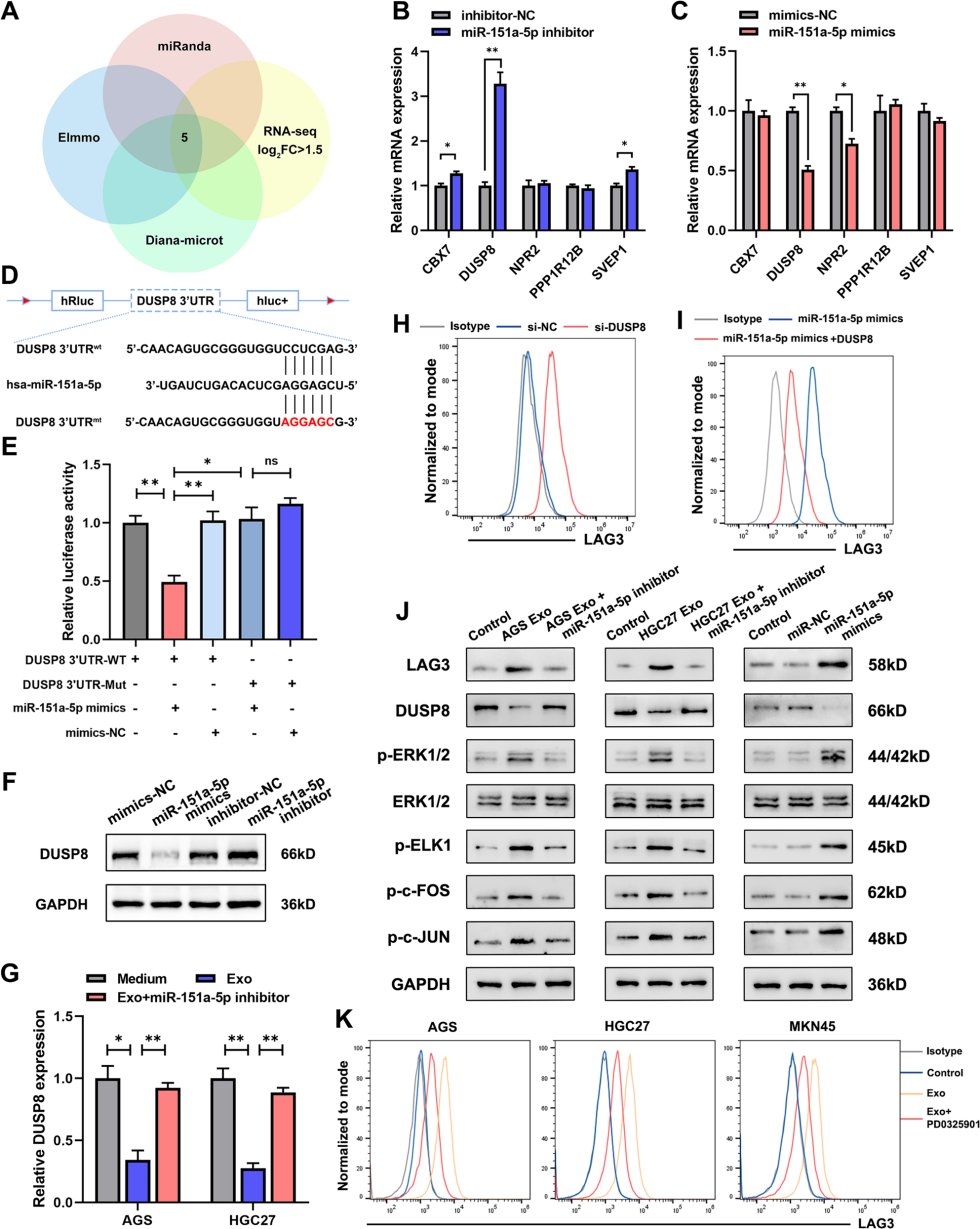
GC-derived exosomal miR-151a-5p regulated DUSP8/MAPK signaling axis in macrophages. **A** Venn plot of RNA sequencing and miR target databases (miRanda, EImmo, Diana-microT). **B-C** Regulatory effect of miR-151a-5p inhibitor and mimics on the expression levels of candidate target genes as detected by qRT-PCR. **D** Schematic diagram of the potential binding sequence between miR-151a-5p and DUSP8. **E** Dual-luciferase reporter assay validating the binding sequence between miR-151a-5p and DUSP8. **F** Western blot of DUSP8 protein levels regulated by miR-151a-5p. **G** qRT-PCR analysis of exosomal miR-151a-5p-mediated regulation of DUSP8 mRNA levels. **H** Flow cytometry was used to assess LAG3 levels after DUSP8 interference. **I** Flow cytometry was used to evaluate the regulation of LAG3 levels in LAG3^+^TAMs after transfection with miR-151a-5p mimic and subsequent DUSP8 overexpression. **J** Western blot of LAG3 and MAPK signaling pathway protein expression in macrophages treated with GC cells-derived exosomes ± miR-151a-5p inhibitor. **K** Flow cytometry analysis of LAG3 expression in LAG3^+^ TAMs treated with GC cells-derived exosomes ± MEK inhibitor PD0325901. All experiments were repeated 3 times with consistent results. The data are presented as the means ± SD. *p*-values were determined by two-tailed unpaired Student’s *t*-test. *p < 0.05, **p < 0.01, “ns” not significant versus the control group

To elucidate the molecular mechanisms underlying miR-151a-5p-mediated regulation in macrophages, we performed transcriptomic sequencing using macrophages treated with miR-151a-5p inhibitor (Fig. S7A). Integrative analysis of RNA-seq data with predictions from miRanda, EImmuno, and DIANA-microt databases identified five potential downstream targets: CBX7, DUSP8, NPR2, PPP1R12B and SVEP1 (Fig. 6A). Subsequent validation via transfection of macrophages with miR-151a-5p mimics or inhibitors revealed that *DUSP8* expression was most consistently and significantly modulated, suggesting it as a primary regulatory target (Fig. 6B-C).

Bioinformatic analysis based on sequence complementarity predicted a putative binding site for miR-151a-5p within the 3′ untranslated region (3′UTR) of DUSP8 (Fig. 6D). This interaction was functionally validated by dual-luciferase reporter assay, which demonstrated that miR-151a-5p mimics significantly reduced luciferase activity from the wild-type DUSP8 3′UTR, but not from a mutant construct (Fig. 6E). Consistent with these findings, miR-151a-5p mimics and inhibitors regulated DUSP8 protein levels in opposite trends (Fig. 6F). Similarly, in macrophages cocultured with GC cells-derived exosomes, DUSP8 mRNA expression was upregulated by the miR-151a-5p inhibitor (Fig. 6G).

To investigate whether miR-151a-5p modulated the function of macrophages through suppression of DUSP8 expression, we performed KEGG and Gene Ontology (GO) enrichment analysis of transcriptomic sequencing data, which implicated the MAPK signaling pathway as a potential downstream target of miR-151a-5p in macrophages (Fig. S7B-C). Flow cytometry analysis revealed that the knockdown of DUSP8 expression in macrophages led to upregulated LAG3 levels (Fig. 6H and Fig. S7D), and the upregulation of LAG3 by miR-151a-5p mimics was abrogated by DUSP8 overexpression (Fig. 6I and Fig. S7E).

DUSP8 belongs to the dual-specificity phosphatase (DUSP) subfamily, which deactivates target kinases primarily by dephosphorylating phosphoserine/threonine and phosphotyrosine residues, with notable activity toward members of the mitogen-activated protein kinase (MAPK) superfamily, including ERK, JNK and ELK1. Given this role, we hypothesized that GC cells-derived exosomal miR-151a-5p regulated LAG3 expression in macrophages via DUSP8-mediated modulation of the MAPK signaling pathway. Western blotting revealed that GC cells-derived exosomes suppressed DUSP8 expression and concomitantly increased phosphorylation of key MAPK pathway components, including ERK1/2, ELK1, c-FOS and c-JUN. In contrast, macrophages transfected with miR-151a-5p inhibitor exhibited restored DUSP8 levels and inhibited MAPK pathway activation, even in the presence of exosomes. Furthermore, miR-151a-5p mimics alone were sufficient to activate the MAPK signaling pathway in macrophages (Fig. 6J). Pharmacological inhibition of the MAPK pathway with PD0325901 attenuated exosome-induced LAG3 upregulation, and a comparable effect was observed in macrophages co-treated with PD0325901 and miR-151a-3p mimics (Fig. 6K and Fig. S8A–C). Moreover, PD0325901 treatment significantly reversed the suppressive effect of miR-151a-3p mimic-transfected LAG3⁺ TAMs on T cell effector function (Fig. S8D–F), providing functional evidence implicating this pathway in TAM-mediated immunosuppression. Together, these findings indicated that exosomal miR-151a-5p promoted LAG3 expression in macrophages through a DUSP8-dependent activation of MAPK signaling.

### ELK1 enhanced LAG3 transcription in LAG3^+^ TAMs

**Fig. 7:**
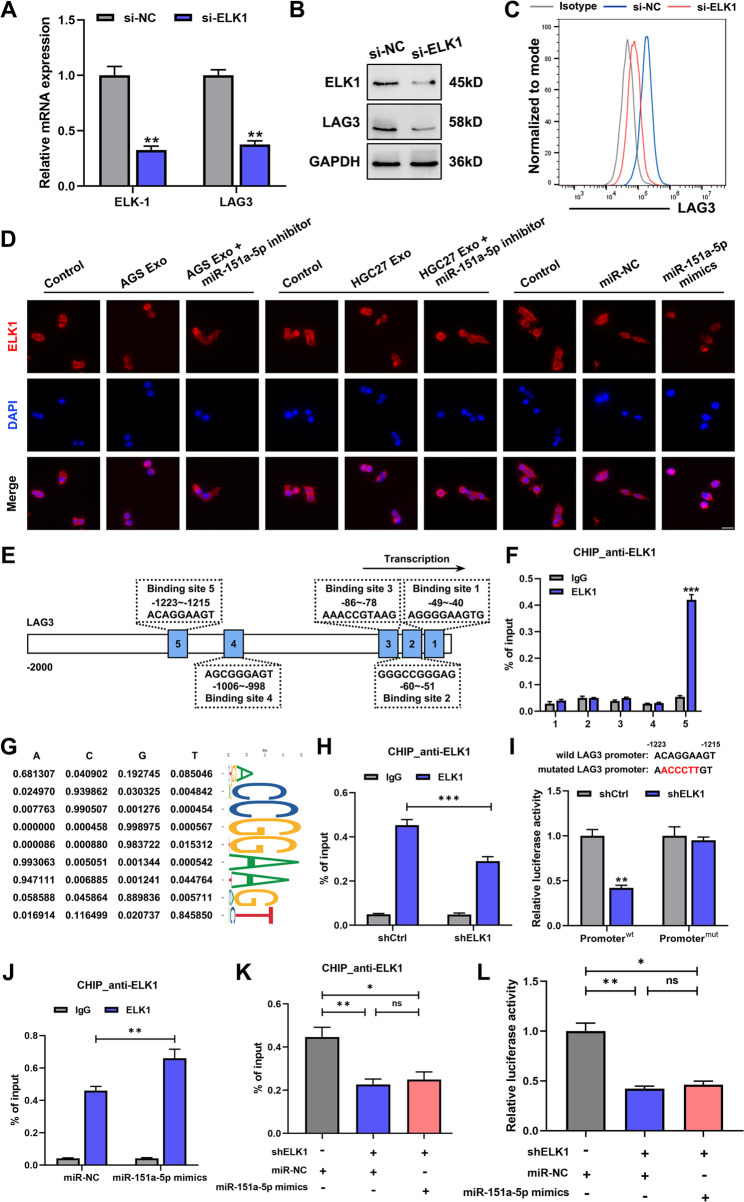
ELK1 enhanced LAG3 transcription in LAG3^+^ TAMs. **A** qRT-PCR analysis of LAG3 mRNA levels in LAG3^+^TAMs following ELK1 knockdown. **B** Western blot analysis of LAG3 protein levels in LAG3^+^TAMs following ELK1 knockdown. **C** Flow cytometry of LAG3 expression in LAG3^+^TAMs following ELK1 knockdown. **D** ELK1 subcellular localization in LAG3^+^TAMs treated with exosome-derived miR-151a-5p examined by Immunofluorescence. **E** Potential ELK1 binding sites in LAG3 promoter predicted by JASPAR database. **F** ChIP-qPCR validation of five potential ELK1 binding sites. **G** Potential sites for the region − 1223 to -1215 (predicted site No.5). **H** ChIP-qPCR analysis of the effect of shELK1 on ELK1 binding to the − 1223 to -1215 region of the LAG3 promoter. **I** Dual-luciferase analysis of ELK1 binding to LAG3 promoter region. **J** ChIP-qPCR assessment of the effect of miR-151a-5p mimics on ELK1 binding to the LAG3 promoter. **K** ChIP-qPCR analysis of the effect of ELK1 knockdown on miR-151a-5p-mediated enhancement of LAG3 transcriptional activation. **L** Effect of ELK1 knockdown on miR-151a-5p-driven LAG3 promoter activity by dual-luciferase assay. All experiments were repeated 3 times with consistent results. The data are presented as the means ± SD. *p*-values were determined by two-tailed unpaired Student’s *t*-test. *p < 0.05, **p < 0.01, ***p < 0.001, “ns” not significant versus the control group

ELK1 is a key transcription factor downstream of the MEK/ERK signaling cascade within the MAPK pathway. Phosphorylation-induced conformational changes in ELK1 enhance its DNA-binding affinity and transcriptional activity. We observed that knockdown of ELK1 in macrophages led to a concomitant reduction in both LAG3 mRNA and protein levels (Fig. 7A-B). Flow cytometry further confirmed a decrease proportion of LAG3^+^ TAMs upon ELK1 silencing, even in the presence of miR-151a-5p (Fig. 7C and Fig.SF9A-B), supporting that miR-151a-5p regulated LAG3 in an ELK1-dependent manner.

To investigate whether ELK1 directly modulates LAG3 transcription, we first assessed its subcellular localization. Immunofluorescence assay demonstrated that miR-151a-5p derived from gastric cancer cell exosomes promoted the nuclear localization of ELK1 in LAG3^+^TAMs, whereas treatment with the miR-151a-5p inhibitor significantly enhanced its retention in the cytoplasm (Fig. 7D). Next, we interrogated the JASPAR database to identify putative ELK1 binding motifs within the LAG3 promoter region, yielding five candidate sequences (Fig. 7E). ChIP-qPCR assay demonstrated that sequence No.5 (-1223 to -1215) was the most significantly enriched, corroborated by its high JASPAR conservation score (Fig. 7F-G). Specificity was confirmed by reduced enrichment of this region upon ELK1 knockdown (Fig. 7H).

Luciferase reporter assays further validated functional engagement. Silencing ELK1 attenuated luciferase activity driven by the wild-type LAG3 promoter, but not by a mutant construct harboring deletions in the No. 5 binding site (Fig. 7I). Furthermore, while miR-151a-5p mimics enhanced the occupancy of the transcription factor ELK1 at the LAG3 promoter, this effect was attenuated by both a miR-151a-5p inhibitor and the application of PD0325901(Fig. 7J and Fig.SF9C-D), suggesting upstream regulation via the miR-151a-5p/DUSP8/MAPK axis. Critically, ChIP-qPCR and Luciferase reporter assay demonstrated that the ability of miR-151a-5p mimics to enhance LAG3 promoter activity was abolished upon ELK1 knockdown (Fig. 7K-L).

These above results confirmed that miR-151a-5p promoted LAG3 expression in macrophages in an ELK1-dependent manner, positioning ELK1 as a direct transcriptional activator of LAG3 downstream of exosomal miR-151a-5p signaling.

### LAG3^+^ TAMs promoted the secretion of GC-derived exosomal miR-151a-5p through the CXCL8/CXCR2 axis

Having established that GC-derived exosomal miR-151a-5p promoted the infiltration of LAG3⁺ TAMs through the DUSP8/MAPK/ELK1 signaling axis, ultimately contributing to T cell exhaustion, we next sought to elucidate the intrinsic molecular mechanisms by which highly infiltrating LAG3⁺ TAMs drive tumor progression in the GC microenvironment. TAMs are known to promote tumorigenesis largely through the secretion of cytokines, chemokines and extracellular matrix components. Among these, cytokines can bind to specific receptors on tumor cells, thereby activating intracellular signaling pathways that modulate malignant behaviors.

**Fig. 8:**
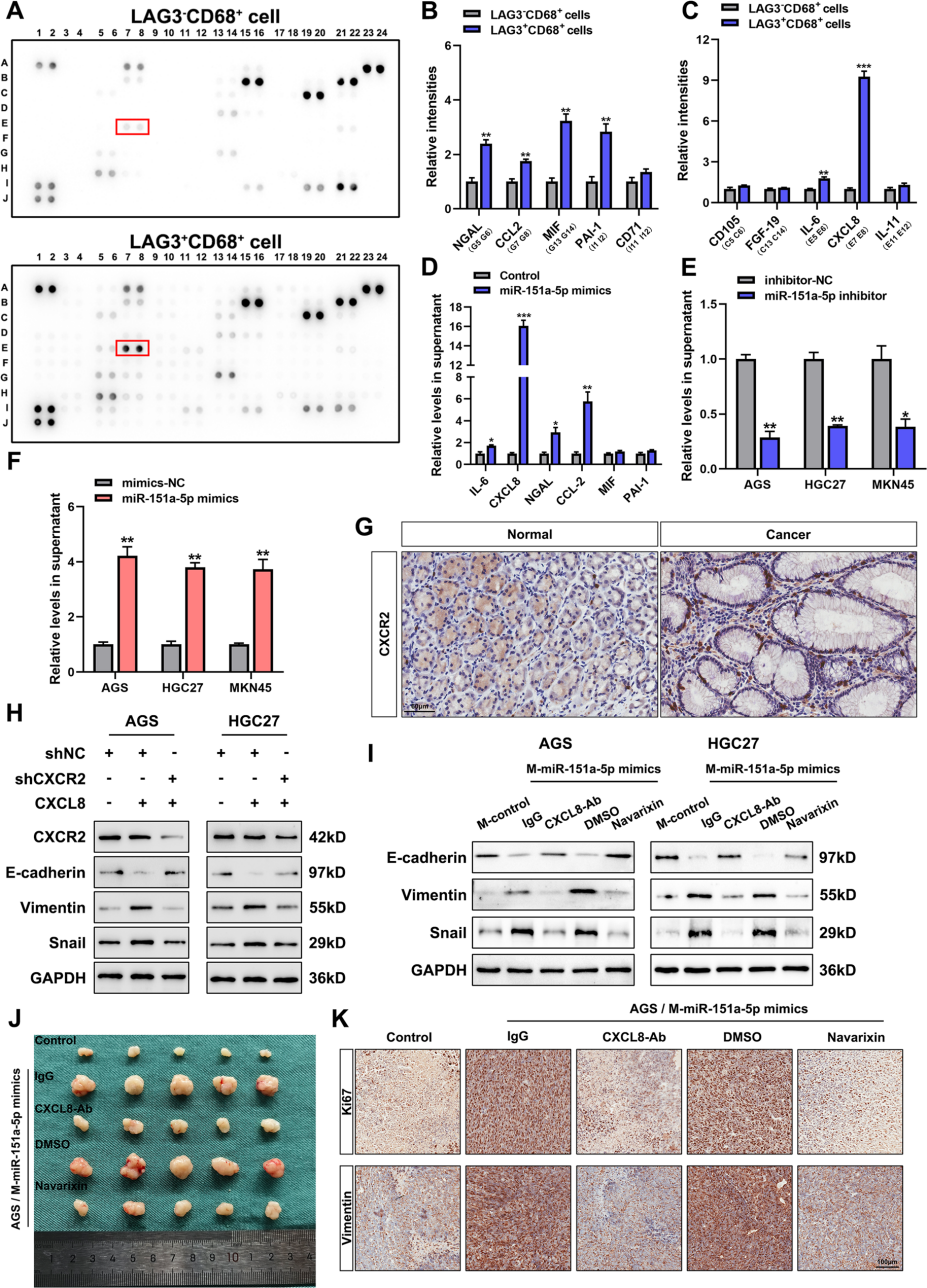
LAG3^+^TAMs promoted the secretion of GC-derived exosomal miR-151a-5p through the CXCL8/CXCR2 axis. **A** Cytokine array analysis of cytokines secreted by LAG3^−^CD68^+^TAMs and LAG3^+^CD68^+^TAMs. **B-C** ELISA validation of cytokine array results. **D** ELISA-based detection of miR-151a-5p mimic-mediated regulation of cytokine secretion. **E-F** ELISA assays to detect the regulation of mir-151a-5p inhibitor and mimics on the secretion of CXCL8. **G** Immunohistochemical of CXCR2 expression in GC and non-tumor tissues. **H** Western blot analysis of E-cadherin, Vimentin, and Snail expression in GC cells following treatment with exogenous CXCL8 and/or known down of shCXCR2. **I** EMT marker levels in GC cells treated with conditioned medium from miR-151a-5p-transfected TAMs ± CXCL8-neutralizing antibody or Navarixin. **J** Subcutaneous transplantation of gastric cancer cells under different treatment conditions in nude mice (*n* = 5). K Immunohistochemical results of Ki67 and vimentin in subcutaneous xenografts. All experiments were repeated 3 times with consistent results. The data are presented as the means ± SD. *p*-values were determined by two-tailed unpaired Student’s *t*-test. *p < 0.05, **p < 0.01, ***p < 0.001 versus the control group

To determine whether LAG3⁺TAMs exert pro-tumorigenic effects via cytokine signaling, we isolated LAG3⁻CD68⁺ TAMs and LAG3⁺CD68⁺TAMs using fluorescence-activated cell sorting. Cytokine profiling of their conditioned supernatants revealed differential secretion of several cytokines, including NGAL, CCL2, MIF, IL-6 and CXCL8 (Fig. 8A-C), with CXCL8 showing the most pronounced upregulation in LAG3⁺TAMs. ELISA assays confirmed that miR-151a-5p promoted CXCL8 secretion in LAG3⁺TAMs (Fig. 8D). Furthermore, exosomes derived from gastric cancer cells, when loaded with miR-151a-5p mimics or inhibitors, were found to correspondingly enhance or reduce CXCL8 secretion in these cells (Fig. 8E-F).

CXCL8, a member of the CXC chemokine family, signals through the G protein-coupled receptor CXCR2 and is implicated in tumor cell proliferation, angiogenesis, immunosuppression and metabolic reprogramming. IHC analysis revealed significantly elevated CXCR2 expression in GC tissues compared to non-tumorous tissues (Fig. 8G), suggesting enhanced responsiveness to CXCL8 in the tumor compartment. We next investigated the functional impact of CXCL8 secreted by LAG3⁺TAMs on GC progression via CXCR2. Exogenous CXCL8 treatment of GC cells upregulated mesenchymal markers (Vimentin, Snail) and downregulated E-cadherin, indicative of epithelial–mesenchymal transition (EMT). Notably, these effects were abrogated upon CXCR2 knockdown (Fig. 8H). Interestingly, we identified that exogenous CXCL8 increased the levels of miR-151a-5p in GC cells-derived exosomes without altering intracellular miR-151a-5p expression, suggesting selective enrichment into exosomes (Fig. S10A-B). Co-culture of GC cells with LAG3⁺TAMs transfected with miR-151a-5p mimics further upregulated EMT marker expression. Conversely, blockade of the CXCL8/CXCR2 axis—using either CXCL8-neutralizing antibodies or the CXCR2 antagonist Navarixin—attenuated EMT marker expression (Fig. 8I) and reduced the loading of miR-151a-5p into GC cell-derived exosomes (Fig. S10C–D).

To validate these findings in vivo, we performed subcutaneous xenograft experiments in nude mice. Tumors derived from GC cells co-cultured with miR-151a-5p-overexpressing LAG3⁺ TAMs exhibited accelerated growth. This pro-tumorigenic effect was significantly attenuated by co-administration of either a CXCL8-neutralizing antibody or Navarixin (Fig. 8J and Fig. S10E). Immunohistochemical analysis of xenograft tumors revealed that co-culture with LAG3⁺TAMs upregulated the expression of Ki67 (the marker of proliferation) and Vimentin (the marker of epithelial-mesenchymal transition), whereas these effects were abrogated upon inhibition of CXCL8 or its receptor, CXCR2 (Fig. 8K and Fig. S10F-G).

### GC cells-derived exosomes shaped the immunosuppressive microenvironment by inducing LAG3⁺ macrophages in vivo

To determine whether GC cells-derived exosomes promote tumor immune evasion through the induction of LAG3⁺TAMs, we established an immune reconstitution model in severely immunodeficient NCG mice. Two weeks after subcutaneous tumor inoculation, we intraperitoneally co-injected purified macrophages and autologous T cells that had been differentially pretreated in vitro (Fig. S11).

**Fig. 9:**
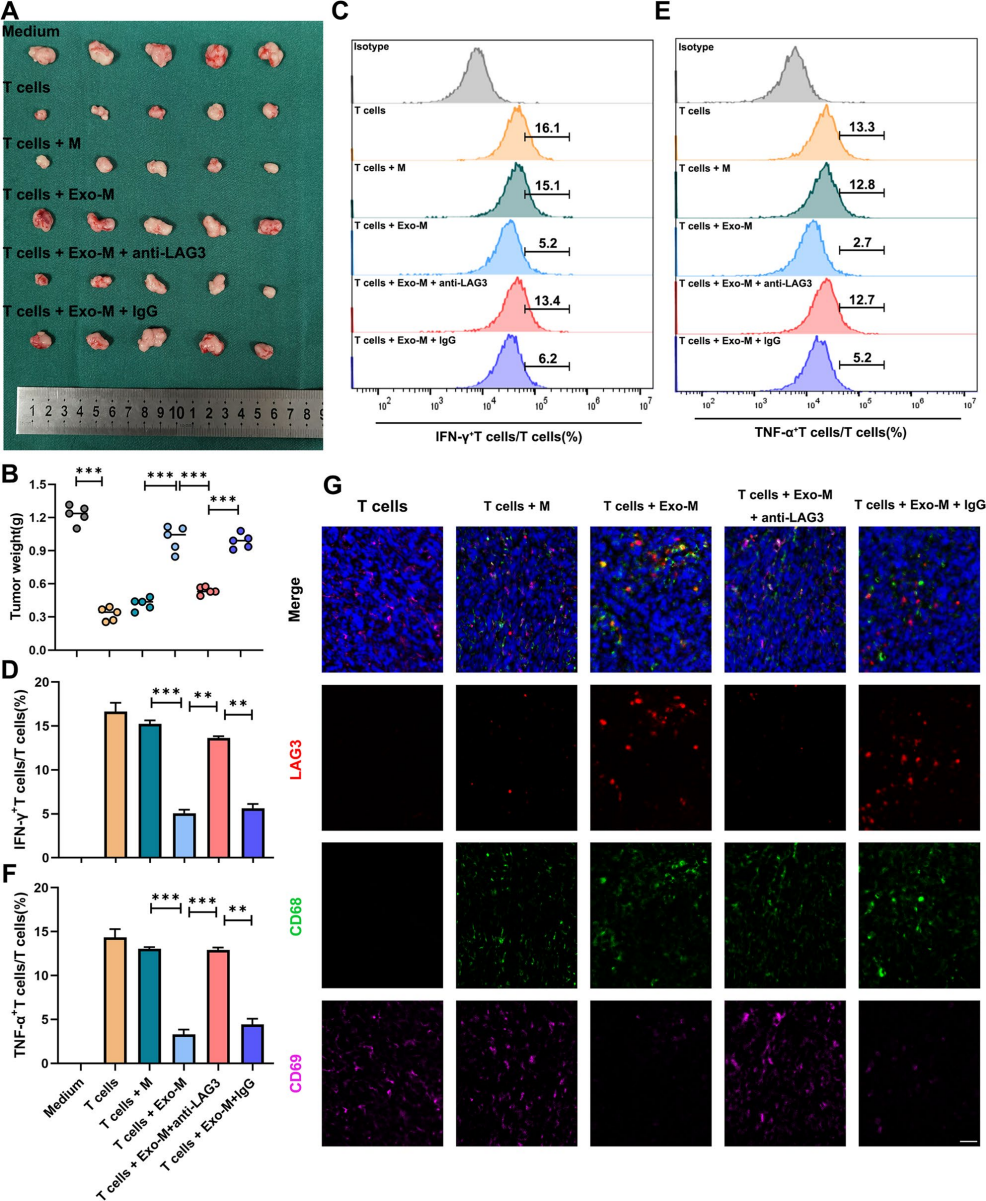
GC cells-derived exosomes shaped the immunosuppressive microenvironment by inducing LAG3⁺TAMs *in vivo.* **A** Subcutaneous transplantation of gastric cancer cells under different treatment conditions in immunodeficient NCG mice (*n* = 5). **B** Tumor weight from the subcutaneous xenograft model in NCG mice. **C-D** Flow cytometry of IFN-γin subcutaneous tumors from NCG mouse models. **E-F** Flow cytometry of TNF-αin subcutaneous tumors from NCG mouse models. **G** mIHC staining of xenograft tumors. All experiments were repeated 3 times with consistent results. The data are presented as the means ± SD. *p*-values were determined by two-tailed unpaired Student’s *t*-test. **p < 0.01, ***p < 0.001 versus the control group

Tumor growth monitoring revealed that macrophages pre-treated with GC cells-derived exosomes significantly impaired T cell–mediated tumor killing. In contrast, administration of a LAG3-neutralizing antibody restored T cell antitumor activity and suppressed tumor progression (Fig. 9A–B). To assess T cell functional status within the TME, we isolated tumor-infiltrating T cells and performed flow cytometry analysis. T cells from mice receiving GC cells-derived exosomes treated macrophages exhibited markedly reduced production of key effector cytokines, including IFN-γ and TNF-α (Fig. 9C–F), indicative of functional exhaustion. mIHC of tumor sections further confirmed that tumors exposed to GC cells-derived exosomes educated macrophages displayed abundant LAG3^+^CD68^+^TAMs infiltration and a concomitant reduction in CD69^+^ activated T cells. Notably, treatment with LAG3-neutralizing antibody reversed this immunosuppressive phenotype, increasing CD69 signal and decreasing the relative abundance of LAG3^⁺^ macrophages in the tumor niche (Fig. 9G).

## Discussion

As understanding of immune checkpoints advances, immune checkpoint inhibitors (ICIs) have become a cornerstone of cancer immunotherapy [33]. By reinvigorating endogenous T-cell responses, ICIs enhance the immune system’s ability to recognize and eliminate tumor cells [34]. The most clinically established targets are the PD-1/PD-L1 axis targeted by nivolumab and pembrolizumab, and CTLA-4 inhibited by agents such as ipilimumab [35]. Despite their clinical success in certain patient populations, key limitations persist—including limited response rates, acquired resistance, and immune-related adverse events (irAEs). To address these challenges, research is increasingly focused on novel checkpoints such as TIM-3, LAG-3 and TIGIT, with therapeutic agents under development [36]. Furthermore, combination strategies, such as dual checkpoint blockade or ICI integration with chemotherapy [37] or anti-angiogenic agents [38] and so on, are being actively investigated to enhance antitumor efficacy through synergistic mechanisms. These approaches aim to broaden T-cell activation and improve clinical outcomes across diverse malignancies. Our findings demonstrate that the emerging immune checkpoint LAG3 exerts immunosuppressive effects within macrophage-mediated tumor immunity, underscoring its potential as a therapeutic target.

Single-cell technologies have revolutionized biomedical research by enabling high-resolution profiling of gene expression, protein expression, and metabolic states at the individual cell level. Among these, CyTOF is a powerful high-throughput platform for single-cell protein analysis [39]. By analyzing high-dimensional protein expression data, CyTOF enables the identification of rare and functionally distinct cell subpopulations within heterogeneous tissues, thereby providing deep insights into cellular heterogeneity and the unique roles of individual cells in both health and disease. For instance, CyTOF has been used to map the cellular architecture of brain metastases, define the blood-tumor interface, and identify a stromal compartment enriched in immunosuppressive T cells and macrophages [40]. In hepatocellular carcinoma, Zhou et al. applied CyTOF to characterize the immune microenvironment of portal vein tumor thrombus (PVTT), revealing significant enrichment of C5aR^+^ TAMs that promote an immunosuppressive TME and correlate with disease progression and poor prognosis [41]. In this study, we performed CyTOF analysis on five matched pairs of GC and adjacent non-tumor tissues to delineate dysregulated immune cell subsets within the TME of GC. Our results revealed widespread alterations in immune cell infiltration. Notably, we identified a previously uncharacterized LAG3^+^ TAM subpopulation that is highly enriched in GC tissues. Clinical evidence demonstrates that the combination of relatlimab (anti-LAG3) and nivolumab (anti-PD-1) has also demonstrated clinically meaningful benefit in patients with gastric or gastroesophageal junction adenocarcinoma (NCT03662659) [42]; however, the underlying molecular mechanisms remain poorly understood. Our findings deepen the understanding of the mechanistic basis of anti-LAG3 immunotherapy in GC and reveal potential cellular targets for novel immunotherapeutic strategies.

TAMs are key components of the TME, exhibiting marked heterogeneity and plasticity [43]. Although macrophages can mediate tumor cell phagocytosis and promote anti-tumor immunity under certain conditions, within the TME they often undergo phenotypic and functional reprogramming that supports tumor progression [44]. TAMs promote tumor invasion, metastasis and angiogenesis through the secretion of MMPs [45] and VEGF [46], suppress anti-tumor immunity by producing IL-10 and TGF-β and upregulating PD-L1 expression [47], synergize with myeloid-derived suppressor cells to further impair immune responses [48], and contribute to tumor progression via metabolic reprogramming. Consequently, modulating TAM polarization or targeting their metabolic pathways has emerged as a promising immunotherapeutic strategy, aiming not only at suppressing their pro-tumorigenic functions but also at reprogramming them into anti-tumor effectors. In this study, we identified that a distinct LAG3^+^ TAM subtype that is highly enriched in the TME of GC. High infiltration of this subtype correlates with functional exhaustion of CD8^+^ T cells, characterized by reduced proliferative capacity, increased apoptosis and diminished secretion of IFN-γ and TNF-α. Using cytokine microarray analysis, we further identified CXCL8 as a key cytokine secreted by LAG3^+^ TAMs. Mechanistically, the CXCL8/CXCR2 axis promotes the secretion of exosomal miR-151a-5p from GC cells.

Exosomes are key mediators of intercellular communication, capable of transmitting bioactive molecules that modulate diverse physiological and pathological processes in recipient cells, including immune regulation, tissue repair, tumorigenesis and neurodegenerative disorders. Their role in tumor immunity is particularly complex and context-dependent. Exosomes can either stimulate antitumor immunity by presenting tumor antigens or activating T cells, or promote immune suppression by reshaping the TME. For example, tumor-derived exosomal PD-L1 inhibits T cell activation, facilitating immune evasion and tumor progression; conversely, blocking exosomal PD-L1 release has been shown to suppress tumor growth, even in models resistant to anti-PD-L1 antibodies [49].Moreover, exosomes can enhance macrophage recruitment and polarization toward immunosuppressive phenotypes, thereby contributing to an immune-tolerant TME [50]. Critically, exosome function is determined by their cellular origin, molecular cargo and target cell type. In this study, through transcriptomic profiling and in vitro co-culture systems, we demonstrate that GC-derived exosomal miR-151a-5p drives the accumulation of a distinct LAG3^+^ TAM subset in the TME. Mechanistically, miR-151a-5p is transferred from GC cells to macrophages via exosomes and promotes LAG3 expression by activating the DUSP8/MAPK/ELK1 signaling axis. Given the complex and interactive nature of the tumor microenvironment, we agree that the regulatory role of miR-151a-5p may extend beyond the cell types examined in this study. It is plausible that this miRNA also modulates the function and differentiation of other key immunosuppressive components, including Treg cells and MDSCs. Investigating these potential regulatory interactions, and the specific downstream targets involved, will be an important focus of our subsequent investigations.

## Conclusions

Collectively, our study demonstrates a reciprocal crosstalk between GC cells and LAG3^+^ TAMs: GC cells-derived exosomal miR-151a-5p drives the accumulation of LAG3^+^TAMs via the DUSP8/MAPK/ELK1 signaling axis. These LAG3⁺TAMs, in turn, secrete CXCL8, which activates CXCR2 on GC cells. This activation not only reinforces the expression of EMT markers but, critically, also enhances the packaging and export of miR-151a-5p into GC cell-derived exosomes, thereby establishing a positive feedback loop. This feed-forward loop amplifies tumor progression and represents a key mechanism of microenvironmental co-evolution in GC.

## Supplementary Information

Supplementary Material 1.

## Data Availability

The data supporting the conclusions of this article have been given in this article and its additional files.
