# Supplementary material for: Gastric cancer cells-derived exosomal miR-151a-5p induces an immunosuppressive microenvironment through promoting LAG3+TAMs infiltration
Source: J Exp Clin Cancer Res. 2026 Apr 1;45:115. doi: 10.1186/s13046-026-03703-9 (PMC13170018; doi:10.1186/s13046-026-03703-9)
Supplement: Supplementary file 1 — Supplementary Material 1. [file 13046_2026_3703_MOESM1_ESM.docx]

**Supplementary materials**

**Supplementary material 1: Supplementary Figures**

**Supplementary Figure 1: High-dimensional characterization of immune cell heterogeneity in GC via CyTOF data.**


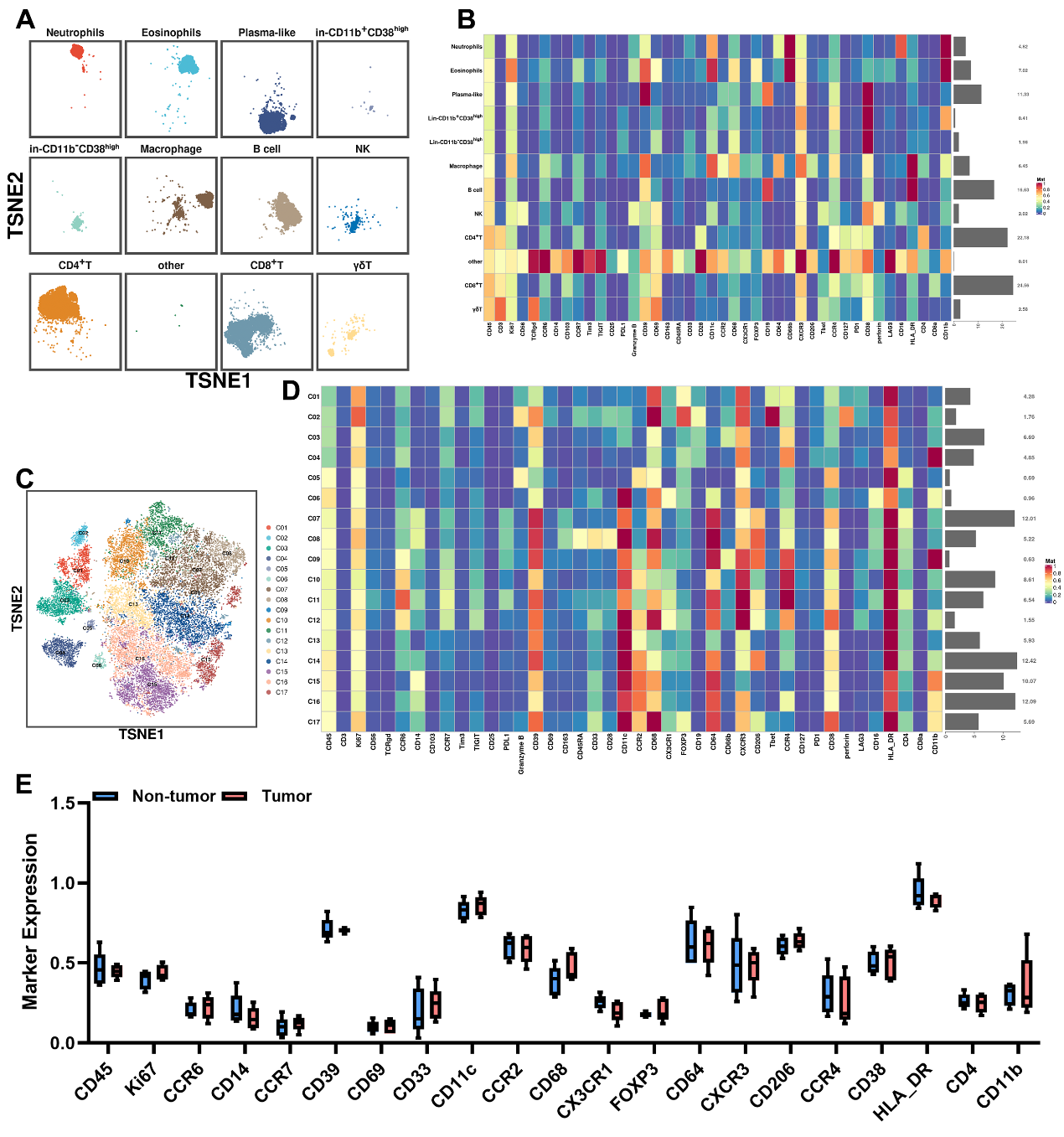


**A** t-SNE visualization of CyTOF data showing immune cell clustering. **B** Heatmap of marker protein expression across immune cell populations. **C** TAMs in GC were clustered into 17 subpopulations. **D** Heatmap of marker protein expression in 17 macrophage subtypes. **E** Cell surface marker expression in C14 macrophage subpopulations (part 2).

**Supplementary Figure 2: Immune regulatory molecule profile of LAG3^+^ TAM in GC.**


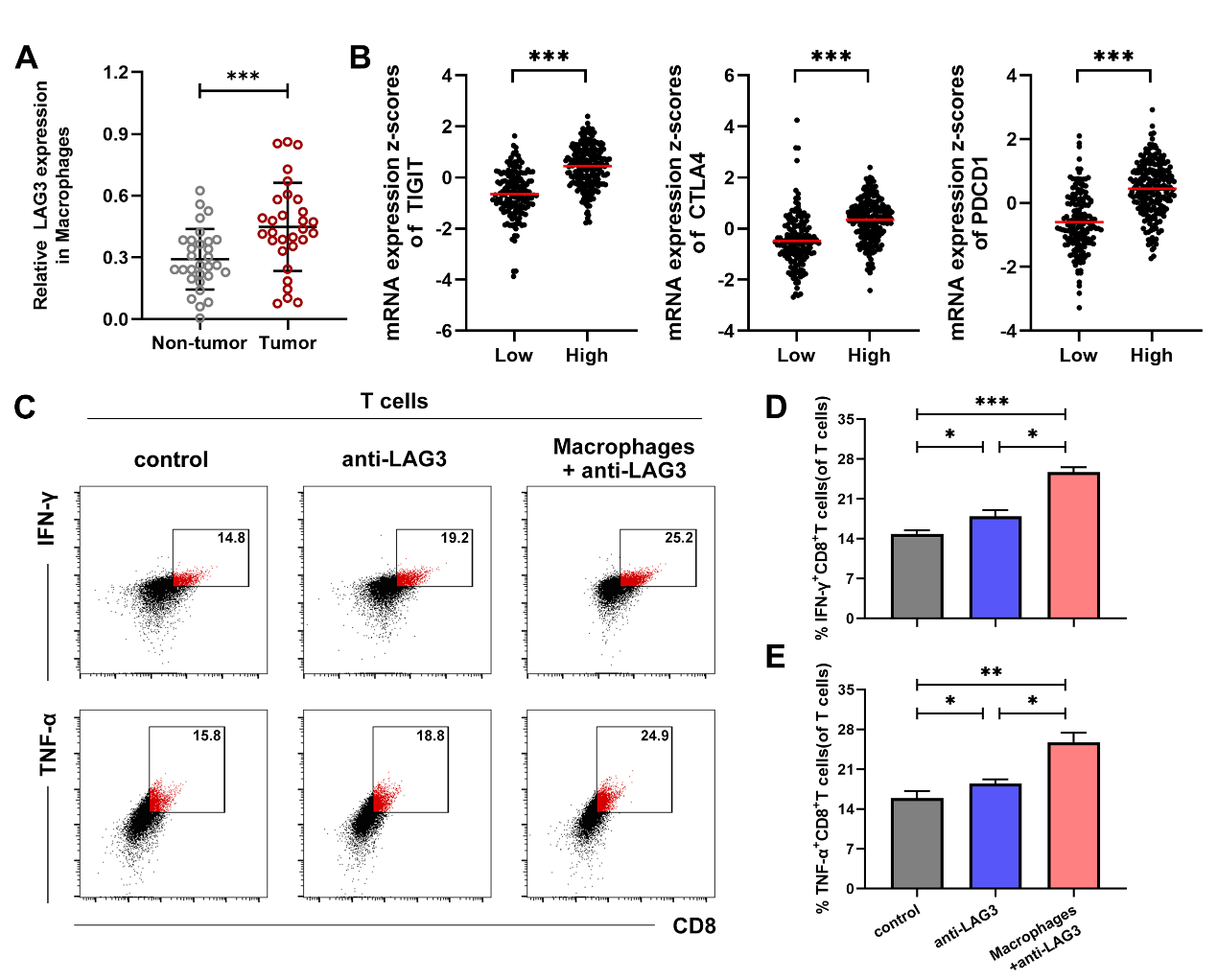
 **A** qRT-PCR analysis of LAG3 expression in TAMs isolated from GC and paired non-tumor tissues n=30. **B** Expression of T cell exhaustion markers (TIGIT, CTLA4, PDCD1) in GC patients stratified by LAG3^+^CD68^+^TAM abundance in the TCGA cohort n^Low^=121, n^High^=221. **C-E** IFN-γ and TNF-α production of T cells after treated with LAG3-neutralizing antibodies±LAG3^+^TAMs. All experiments were repeated 3 times with consistent results. The data are presented as the means ± SD. *p*-values were determined by two-tailed unpaired Student’s *t*-test. **p <* 0.05, ***p <* 0.01, and ****p <* 0.001 versus the control group.

**Supplementary Figure 3: HGC27-derived exosomes promoted LAG3^+^TAM infiltration and mediated T cell suppression.**


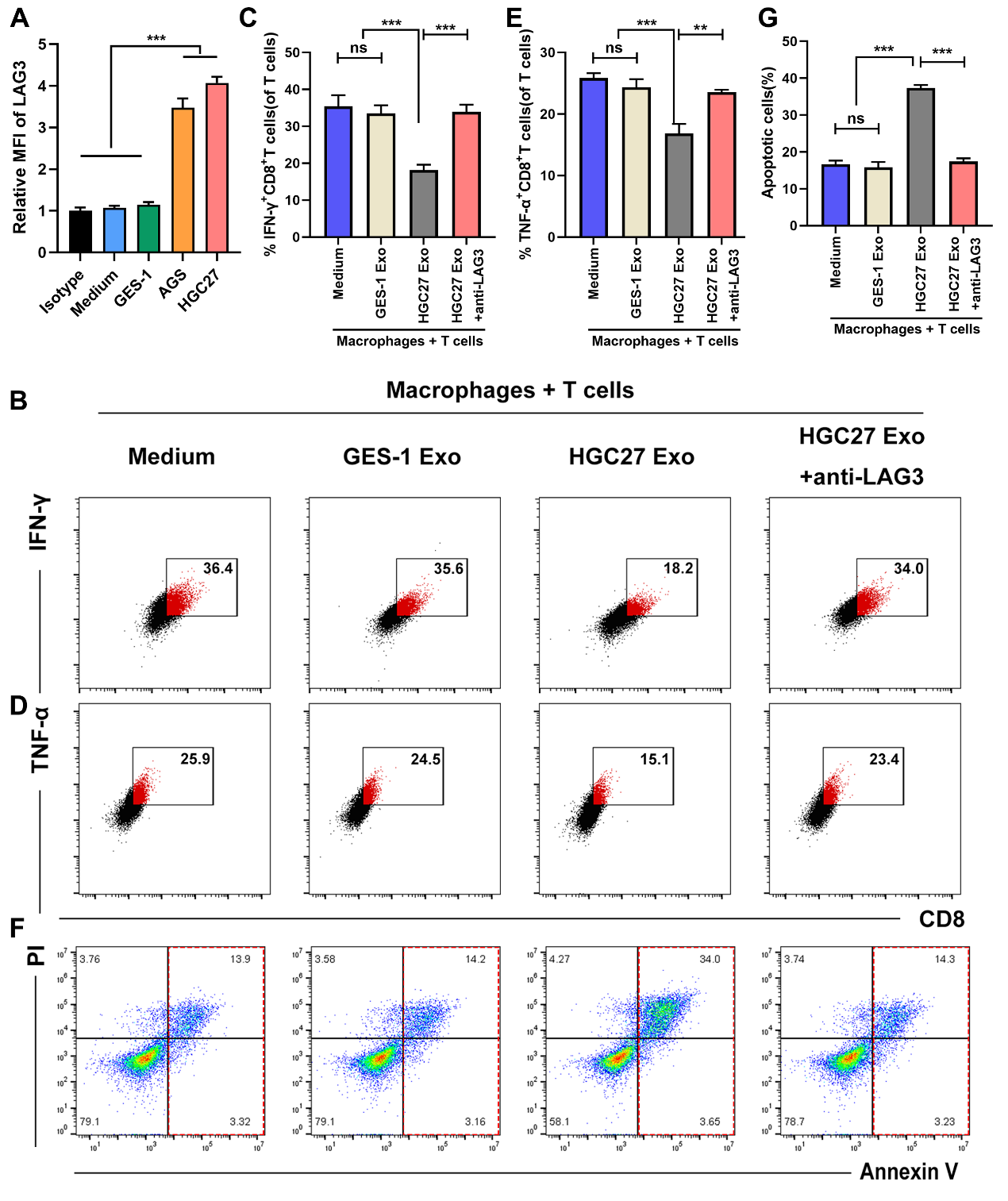


**A** Relative MFI of LAG3 detected in TAMs treated with exosomes derived from GES-1 and GC cells. **B-C** IFN-γ production by T cells co-cultured with HGC27-derived-exosomes treated LAG3^+^ TAMs ± LAG3-neutralizing antibodies. **D-E** TNF-α production by T cells co-cultured with HGC27-derived-exosomes treated LAG3^+^TAMs ± LAG3-neutralizing antibodies. **F-G** Apoptosis levels of T cells co-cultured with HGC27-derived-exosomes treated LAG3^+^TAMs ± LAG3-neutralizing antibodies. All experiments were repeated 3 times with consistent results. The data are presented as the means ± SD. *p*-values were determined by two-tailed unpaired Student’s *t*-test. ***p <* 0.01, ****p <* 0.001, “ns” not significant versus the control group.

**Supplementary Figure 4:** **Expression of mir-151a-5p in gastric cancer patients**


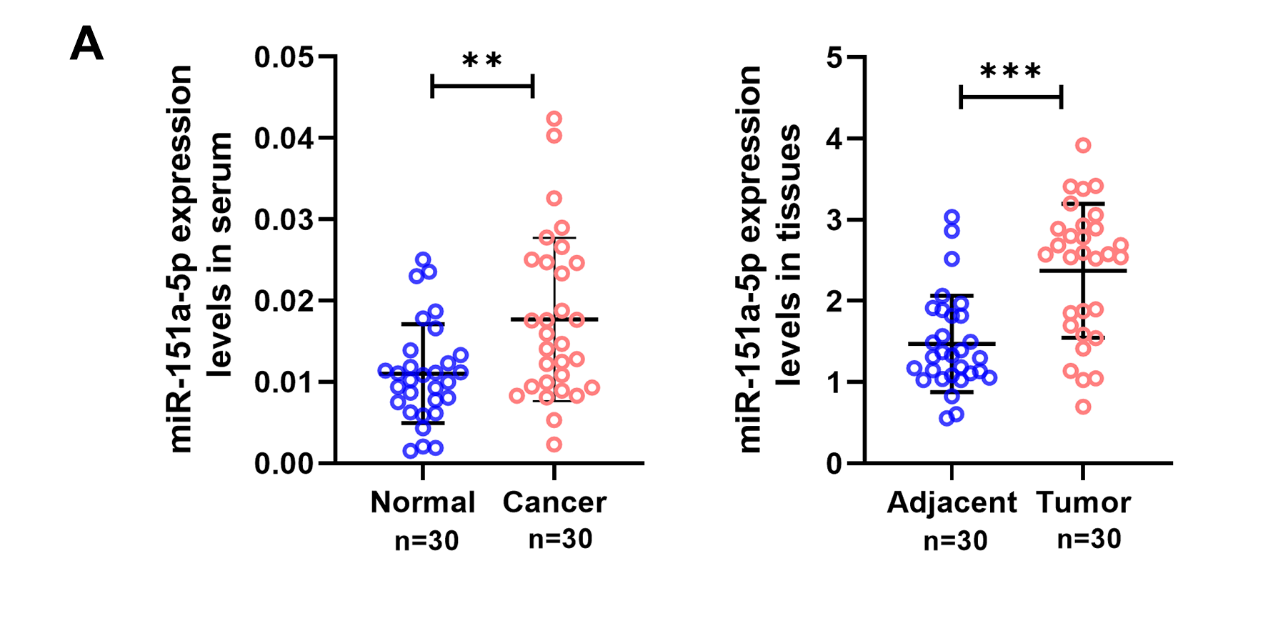


A Expression of mir-151a-5p in gastric cancer serum samples n=30. B Expression of mir-151a-5p in gastric cancer tissues samples n=30. The data are presented as the means ± SD. *p*-values were determined by two-tailed unpaired Student’s *t*-test. ***p <* 0.01, ****p <* 0.001 versus the control group.

**Supplementary Figure 5: The regulatory effect of miR-151a-5p on LAG3 expression.**


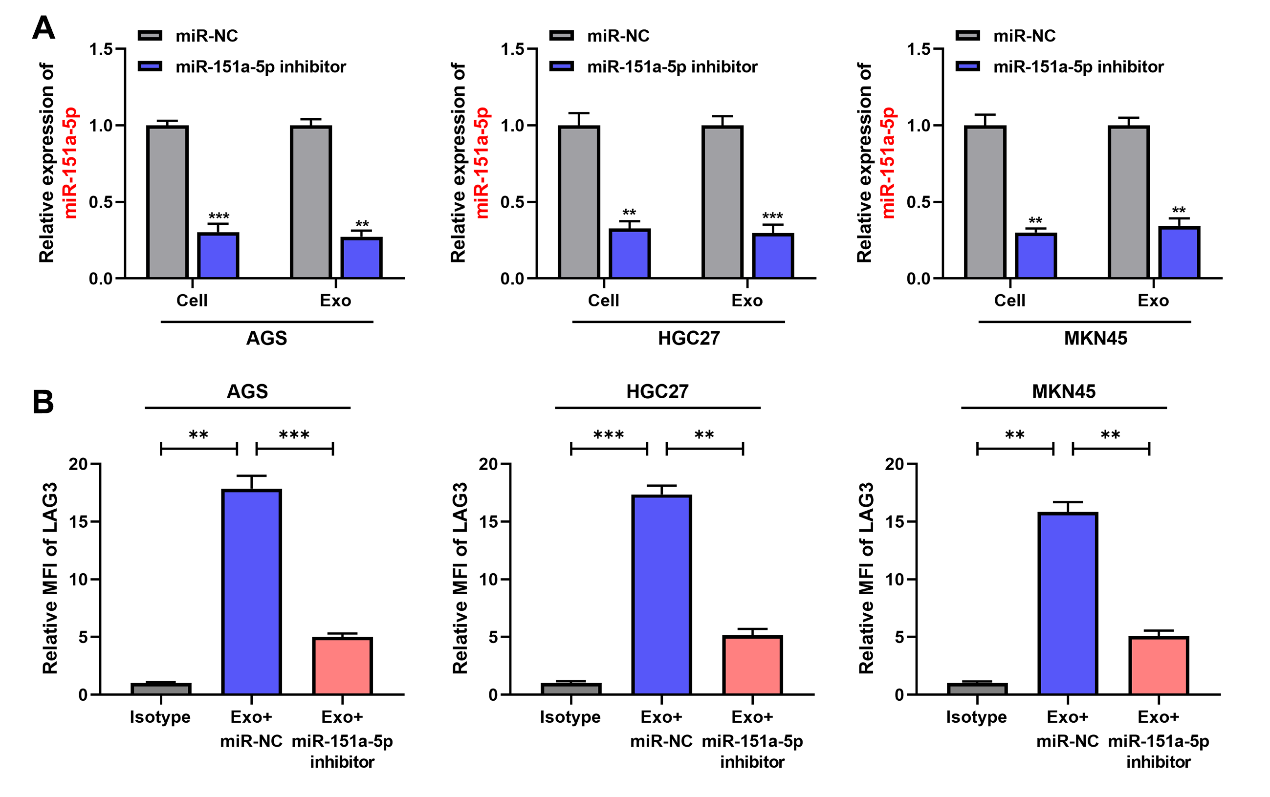
 **A** Validation of the efficiency of the miR-151a-5p inhibitor in GC cells. **B** Relative MFI of LAG3 in LAG3^+^ TAMs after co-incubation with exosomes from GC cells transfected with miR-NC or miR-151a-5p inhibitor. All experiments were repeated 3 times with consistent results. The data are presented as the means ± SD. *p*-values were determined by two-tailed unpaired Student’s *t*-test. ***p <* 0.01, and ****p <* 0.001 versus the control group.

**Supplementary Figure 6: Regulatory effect of exosomal miR-151a-5p from GC cells on T cell function through modulation of LAG3^+^ TAMs.**


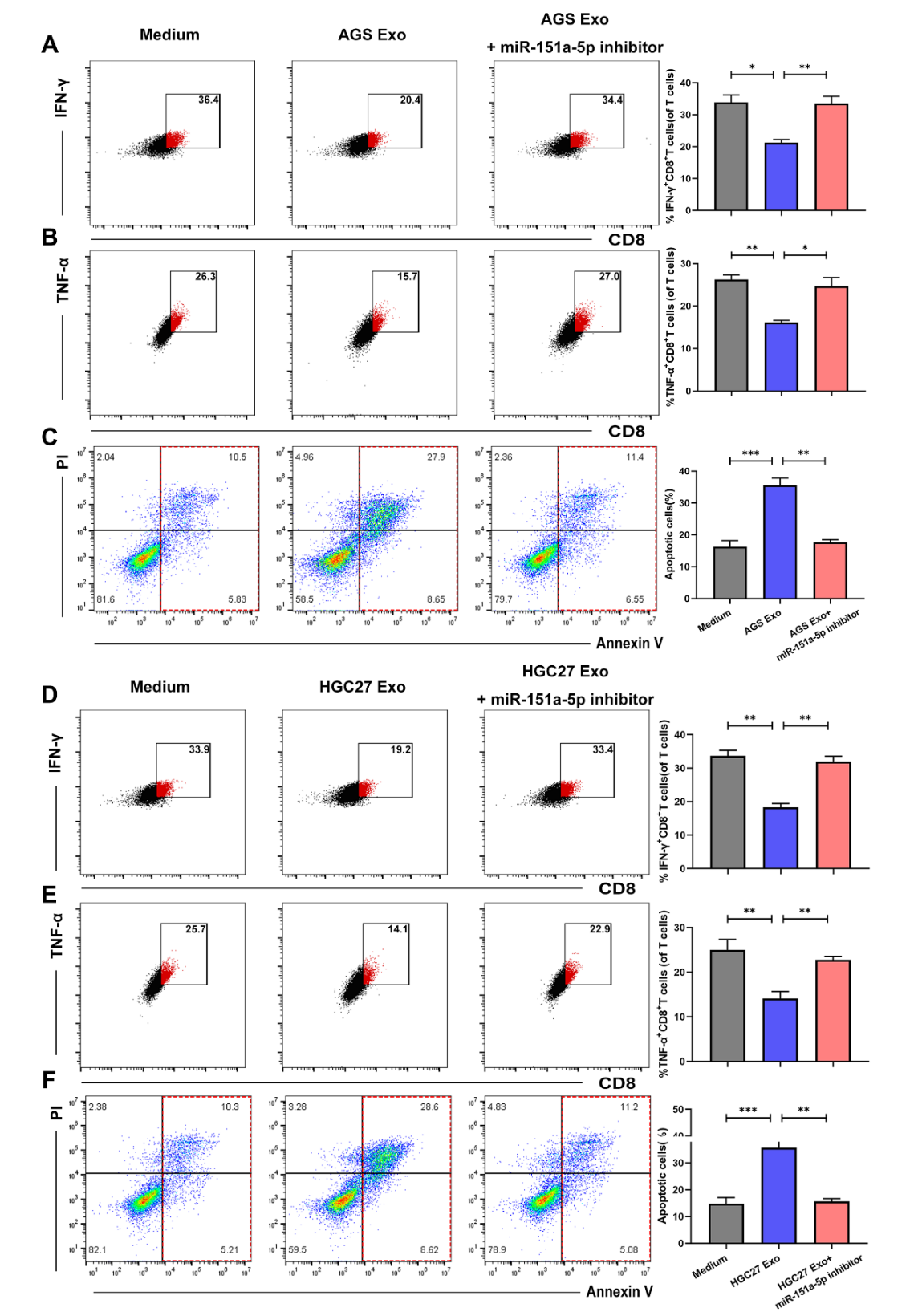


**A-C** IFN-γ, TNF-α, and apoptosis levels in T cells co-cultured with AGS-derived-exosomes treated LAG3^+^TAMs ± miR-151a-5p inhibitor. **D-F** IFN-γ, TNF-α, and apoptosis levels in T cells co-cultured with HGC27-derived-exosomes treated LAG3^+^TAMs ± miR-151a-5p inhibitor. All experiments were repeated 3 times with consistent results. The data are presented as the means ± SD. *p*-values were determined by two-tailed unpaired Student’s *t*-test. **p <* 0.05, ***p <* 0.01, and ****p <* 0.001 versus the control group.

**Supplementary Figure 7: Transcriptomic profiling of miR-151a-5p-mediated regulation in macrophages identifies key signaling pathways and target genes.**


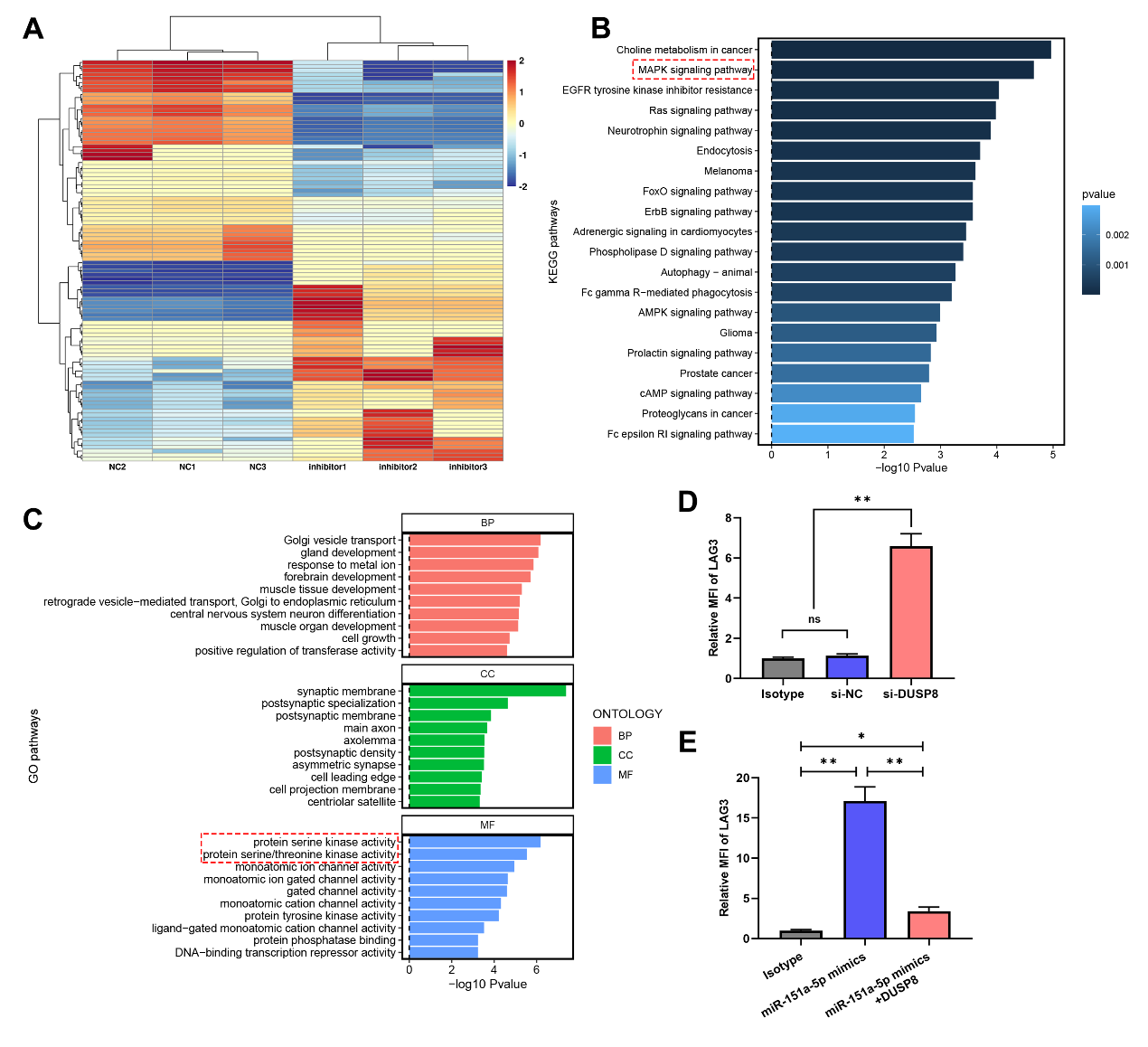


**A** Heatmap of transcriptomic sequencing results from LAG3^+^TAMs transfected with miR-151a-5p inhibitor and miR-NC. **B** KEGG pathway enrichment analysis of RNA-seq data. **C** GO enrichment analysis of RNA-seq data. **D** Relative MFI of LAG3 in LAG3^+^ TAMs after DUSP8 interference.

**E** Relative MFI of LAG3 levels in LAG3^+^ TAMs after transfection with miR-151a-5p mimic and subsequent DUSP8 overexpression. All experiments were repeated 3 times with consistent results. The data are presented as the means ± SD. *p*-values were determined by two-tailed unpaired Student’s *t*-test. **p <* 0.05, ***p <* 0.01, “ns” not significant versus the control group.

**Supplementary Figure 8: The impact of the DUSP-MAPK axis on TAM infiltration and function**


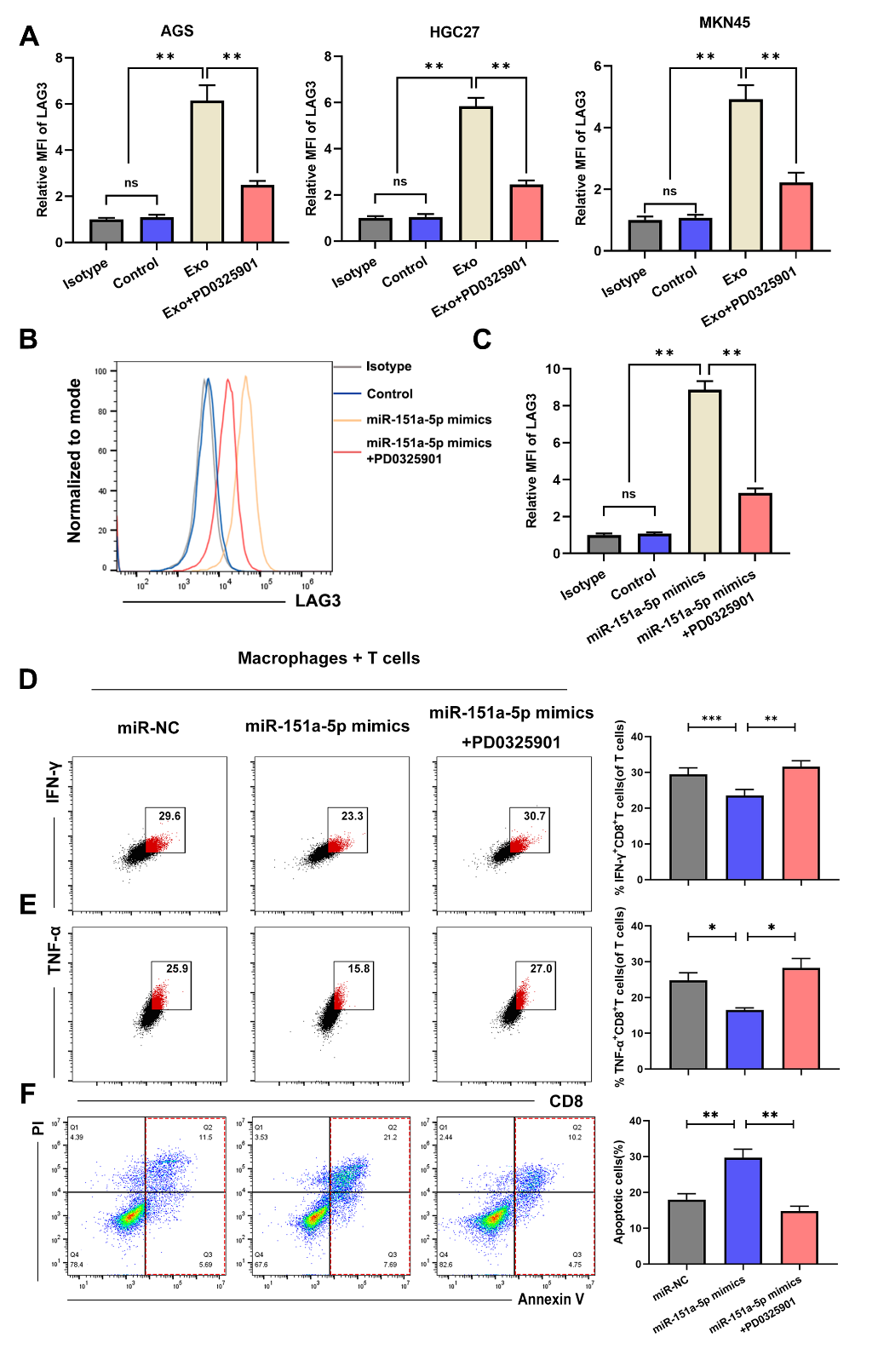


**A** Relative MFI of LAG3 levels in LAG3^+^ TAMs treated with GC-derived exosomes ± PD0325901.

**B** Flow cytometry of LAG3 expression in LAG3^+^TAMs treated with miR-151a-5p inhibitor ± PD0325901. **C** Relative MFI of LAG3 expression in LAG3^+^TAMs treated with miR-151a-5p inhibitor ± PD0325901. **D** IFN-γ, TNF-α, and apoptosis levels in T cells co-cultured with LAG3^+^TAMs treated with miR-151a-5p inhibitor ± PD0325901.All experiments were repeated 3 times with consistent results. The data are presented as the means ± SD. *p*-values were determined by two-tailed unpaired Student’s *t*-test. **p <* 0.05, ***p <* 0.01, ****p <* 0.001, “ns” not significant versus the control group.

**Supplementary Figure 9:** **ELK1 enhanced LAG3 transcription in LAG3^+^ TAMs.**


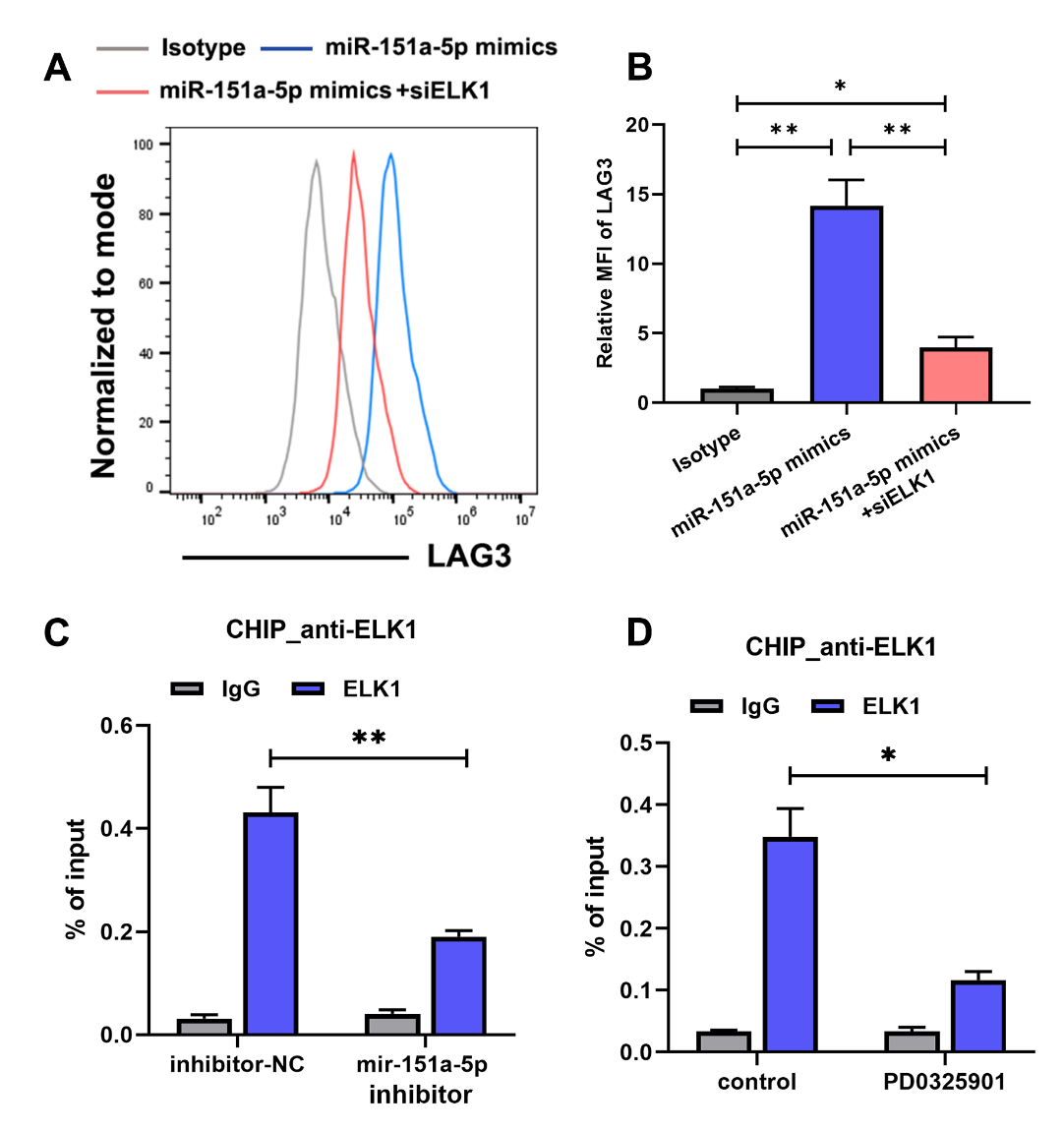


**A** Flow cytometry of LAG3 expression in LAG3^+^TAMs treated with miR-151a-5p mimics ± interference of ELK1. **B** Relative MFI of LAG3 expression in LAG3^+^TAMs treated with miR-151a-5p mimics ± interference of ELK1.**C** ChIP-qPCR assessment of the effect of miR-151a-5p inhibitor on ELK1 binding to the LAG3 promoter. **D** ChIP-qPCR assessment of the effect of PD0325901 on ELK1 binding to the LAG3 promoter. All experiments were repeated 3 times with consistent results. The data are presented as the means ± SD. *p*-values were determined by two-tailed unpaired Student’s *t*-test. **p <* 0.05, ***p <* 0.01 versus the control group.

**Supplementary Figure 10: miR-151a-5p modulation by CXCL8/CXCR2 in GC.**


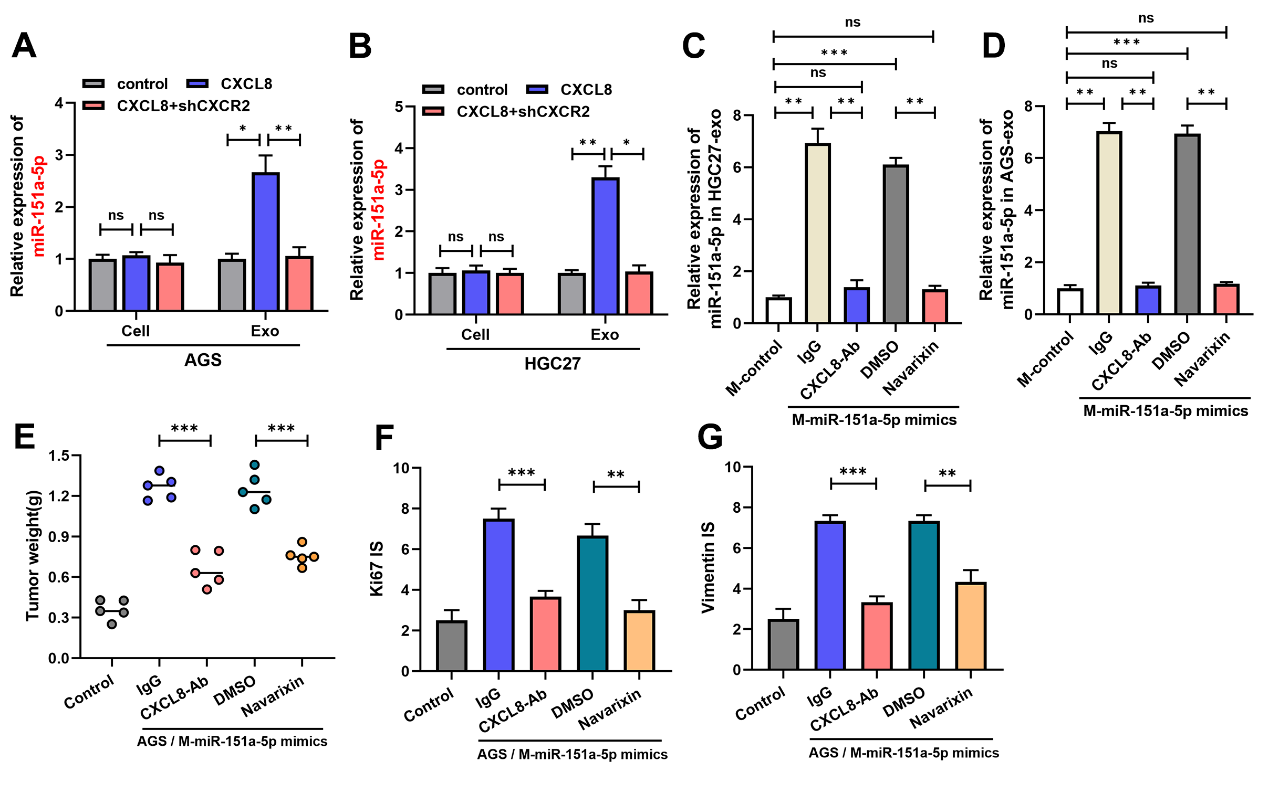
 **A** Effect of CXCL8/CXCR2 on miR-151a-5p expression in AGS cells. **B** Effect of CXCL8/CXCR2 on miR-151a-5p expression in HGC27 cells. **C-D** Relative expression of exosomal miR-151a-5p secretion from GC cells when co-cultured with miR-151a-5p-transfected TAMs ± CXCL8-neutralizing antibody or Navarixin. **E** Tumor weight from the subcutaneous xenograft model in nude mice n=5. **F-G** Immunohistochemical score of Ki67 and Vimentin in xenograft tumors. All experiments were repeated 3 times with consistent results. The data are presented as the means ± SD. *p*-values were determined by two-tailed unpaired Student’s *t*-test. **p <* 0.05, ***p <* 0.01, ****p <* 0.001, “ns” not significant versus the control group.

**Supplementary Figure 11: Overview of the experimental design and procedures.**


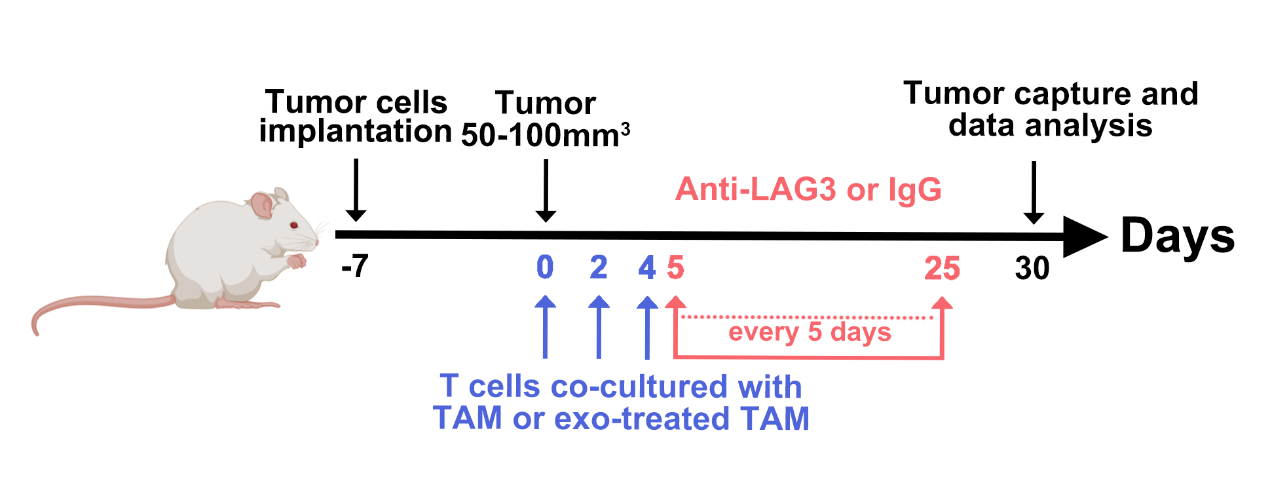


**Supplementary material 2: Supplementary methods**

**Patient Specimens**

Fresh-frozen GC and matched adjacent non-tumor tissues used for CyTOF, Multiplex immunohistochemistry(mIHC) and flow cytometry were obtained from GC patients undergoing surgery resection at the Department of General Surgery, Wuxi No.2 People's Hospital. Immediately after surgical resection and tissue dissociation, samples were snap-frozen in liquid nitrogen and stored for subsequent analysis. All tissue specimens were histopathologically confirmed by two independent pathologists according to the criteria of the 8th AJCC edition. None of the patients had received preoperative chemotherapy, radiotherapy, or immunotherapy prior to surgery. This study was approved by Ethics Committee of the Wuxi No.2 People's Hospital (Approval No. Y-132), and written informed consent was obtained from all participants.

**Cell culture and Transfection**

The human GC cell lines AGS, MKN45, HGC27, as well as the monocytic cell line THP-1 and the T-cell line E6-1 employed in this study, were purchased from KeyGEN BioTECH (Nanjing, China). All cell lines were cultured in RPMI-1640 (Gibco, USA) supplemented with 10% Fetal Bovine Serum (FBS, Gibco, USA) and 1% Penicillin/Streptomycin (P/S) (Gibco, USA) in an incubator at 37 °C in a humidified atmosphere containing 5% CO₂. To induce differentiation of THP-1 cells into macrophage-like cells, 1 × 10⁶ cells/mL were treated with 100 ng/mL phorbol 12-myristate 13-acetate (PMA; Merck, USA) for 24 h. Following differentiation, PMA-containing medium was replaced with fresh complete medium, and cells were allowed to rest for an additional 24 h prior to experimentation. Transient transfection of plasmids constructs, miRNA mimics or miRNA inhibitors (GenePharma, Shanghai, China) were performed using Lipofectamine 3000 or Lipofectamine RNAiMAX (Invitrogen, USA). Transfected cells were harvested 48–72 h post-transfection for downstream assays.

**Exosome Isolation from GC cells**

Exosomes were isolated from the supernatants of GC cell cultures grown in exosome-depleted fetal bovine serum (Exosome-Depleted FBS; System Biosciences, USA). Conditioned media were collected and sequentially centrifuged at 300 × *g* for 10 min, 2,000 × *g* for 20 min, and 10,000 × *g* for 30 min at 4 °C to remove intact cells, cellular debris and large extracellular vesicles or aggregates. The resulting supernatant was then subjected to high-speed centrifugation at 10,000 × *g* for 60–90 min at 4 °C (Beckman Coulter, USA) to pellet medium-sized extracellular vesicles, including exosomes. The pellet was washed with cold phosphate-buffered saline (PBS), resuspended in PBS, and stored at –80 °C for downstream applications. As an alternative method, exosomes were also isolated using the Total Exosome Isolation Reagent (Thermo Fisher Scientific, USA) according to the manufacturer’s instructions. Briefly, the reagent was added to the clarified conditioned medium, followed by incubation at 4 °C for 12–16 h and centrifugation at 10,000 × *g* for 60 min to precipitate exosomes. The resulting pellet was resuspended in PBS and stored at –80 °C.

**Exosome Isolation from GC tissues**

For tissue-derived exosome isolation, fresh gastric cancer tissue specimens were minced into small pieces and incubated in DMEM containing collagenase type I at 37°C for 1 hour with gentle agitation. The resulting cell suspension was sequentially centrifuged at 300 × g for 10 minutes to remove cells, 2000 × g for 20 minutes to eliminate dead cells, and 16,500 × g for 30 minutes to remove large extracellular vesicles and debris. The supernatant was filtered through a 0.22 μm filter and subjected to ultracentrifugation at 110,000 × g for 90 minutes at 4°C using an SW-28 rotor (Beckman Coulter). The crude exosome pellet was resuspended in PBS and layered on top of an iodixanol density gradient (5%, 10%, 20%, and 40%) prepared in PBS, followed by ultracentrifugation at 100,000 × g for 16 hours at 4°C. The exosome-enriched fraction corresponding to densities of 1.09-1.15 g/mL was collected, diluted in PBS, and pelleted by a final ultracentrifugation step at 110,000 × g for 90 minutes. The purified exosomes were resuspended in PBS and stored at -80°C until further analysis

**Exosome Isolation from serum samples**

For serum-derived exosome isolation, peripheral blood samples were collected and centrifuged at 3000 × g for 15 minutes at 4°C to obtain serum. To remove cellular debris and large vesicles, serum was subjected to sequential centrifugation at 2000 × g for 20 minutes and 12,000 × g for 30 minutes at 4°C. The clarified supernatant was then mixed with ExoQuick exosome precipitation solution (System Biosciences) at a 4:1 ratio and incubated overnight at 4°C. The mixture was centrifuged at 1500 × g for 30 minutes at 4°C, and the exosome pellet was resuspended in PBS.

**Exosome Identification**

Isolated exosomes were characterized prior to downstream experiments to confirm their size, morphology, and molecular composition. The hydrodynamic diameter and particle concentration of exosomes were determined using nanoparticle tracking analysis (NTA) with a NanoSight NS300 instrument (Malvern Panalytical, UK). This system tracks and analyzes the Brownian motion of individual particles in suspension, providing size distribution profiles and concentration estimates. Morphological analysis was performed by transmission electron microscopy (TEM). Morphological analysis was performed by transmission electron microscopy (TEM). Briefly, purified exosomes were fixed with 4% glutaraldehyde for 2 h at 4 °C, washed three times with PBS and then adsorbed onto carbon-coated copper grids by droplet deposition. Samples were stained with 2% uranyl acetate for 1 min, air-dried, and imaged under an electron microscope at 80–120 kV. Images were captured with optimized contrast to visualize intact, cup-shaped vesicles within the expected size range of 50–150 nm. To confirm exosomal identity and assess purity, Western Blotting was performed to detect canonical exosomal marker proteins, including CD63, CD9, HSP70 and TSG101. The absence of calnexin, a marker of endoplasmic reticulum and cellular contaminants, was used to verify minimal cellular debris or organelle co-isolation, thereby confirming exosome purity.

**Blockade of exosome generation by GW4869**

To block exosome generation, GW4869 (Sigma, USA), an inhibitor of exosome biogenesis and release, was used. GW4869 was first dissolved in DMSO to prepare a 5mM stock solution, which was then diluted in culture supernatant to achieve a final concentration of 20μM in the cell culture medium (resulting in a final DMSO concentration of 0.005%). To control for potential solvent effects, gastric cancer cells were cultured in 10% exosome-depleted FBS medium supplemented with either GW4869 or an equivalent volume of DMSO (vehicle control). After 72 hours, the conditioned medium was collected and subsequently applied to macrophage cultures.

**Exosomal miRNA Protection Assay**

To confirm that mir-151a-5p was secreted within GC cells-derived exosomes, purified exosomes isolated from culture supernatants were subjected to differential treatments. Equal aliquots of exosomes were incubated at 37°C for 30 min under three conditions: (1) PBS control, (2) RNase A alone (10 μg/mL) to digest unprotected RNAs, and (3) RNase A combined with 1% Triton X-100 to disrupt the exosomal lipid bilayer and expose internal mir-151a-5p to degradation. Following treatment, RNase activity was immediately inhibited, total RNA was extracted using TRIzol reagent, and the levels of mir-151a-5p were quantified by quantitative real-time PCR.

**Flow Cytometry**

For tumor tissue or mouse samples, we initially processed them into single-cell suspensions through grinding, enzymatic digestion, filtration and centrifugation. Subsequently, Fc receptor blocking was performed using anti-CD16/CD32 antibodies (BioLegend, USA) and dead cells were excluded using the Zombie Red Fixable Viability Kit (BioLegend, USA). Cells treated differently were incubated with specific antibodies (Abcam, USA) at 4°C for 30 min, washed twice with PBS, and then analyzed by flow cytometry (CytoFLEX S Flow Cytometer, Beckman Cytometer, USA).

Before detecting cytokines in T cells, 500 ng/mL phorbol myristate acetate (MCE, USA), 1 mg/mL ionomycin (MCE, USA), and 5 mg/mL Brefeldin A (MCE, USA) were used to stimulate the production of cytokines and block secretory for 5 h at 37°C, 5% CO_2_. Next, cells were permeabilized on ice using BD Cytofix/Cytoperm Fixation/Permeabilization Solution Kit (BD Biosciences, USA) and washed twice with Perm/Wash buffer (BD Biosciences, USA). Finally, cells were incubated at 4°C for 30 min with specific antibodies, detected by flow cytometry (CytoFLEX S flow cytometer, Beckman Cytometry, USA) and then analyzed with the FlowJo software.

**Quantitative Real-time polymerase chain reaction (qRT-PCR)**

To extract total RNA, TRIzol reagent was added to actively growing cells, which were then collected in a centrifuge tube and lysed for 10 minutes at room temperature. Subsequently, 200 μl of chloroform wass added per 1 ml of TRIzol, the mixture was vigorously shaken and incubated for 2-3 minutes at room temperature. After centrifugation at 12,000 g for 15 minutes at 4°C, the upper aqueous phase was transferred to a new EP tube. Then, 500 μl of isopropanol was added per 1 ml of TRIzol, the mixture was inverted to mix, incubated for 10 minutes at room temperature and centrifuged again at 12,000 g for 10 minutes at 4°C to obtain the RNA precipitate. The obtained RNA was quantified and then reverse transcribed using reverse transcriptase (Takara-PrimeScript RT reagent Kit, Takara, Japan) for cDNA. QuantiNova SYBR Green PCR Kit (QIAGEN, German) was used for qRT-PCR analysis and GAPDH was selected as an internal reference to calculate the relative expression using the 2^-ΔΔCT^ method. The specific primers applied were mainly exhibited in Table 1.

**Western Blotting**

Tissues or cells were lysed on ice for 20 min using RIPA buffer (CST, USA) supplemented with protease and phosphatase inhibitors (CST, USA). Lysates were sonicated to shear genomic DNA and disrupt cellular aggregates, followed by centrifugation at 14,000 × *g* for 10 min at 4 °C. The resulting supernatants were collected, and total protein concentration was determined using the BCA Protein Assay Kit (Beyotime, China) according to the manufacturer’s instructions. Depending on the target proteins, appropriate SDS-PAGE gels were selected for electrophoresis. The separated proteins were then transferred onto polyvinylidene fluoride (PVDF) membranes (Millipore, USA), which had been pre-activated with methanol. The PVDF membrane was blocked using QuickBlock Blocking Buffer (Beyotime, China) for 1 hour at room temperature to prevent non-specific antibody binding. After blocking, membranes were incubated with primary antibodies (specific for target proteins) at 4°C overnight with gentle agitation. The next day, membranes were washed three times with TBST (5 minutes per wash) and then incubated with the corresponding HRP-conjugated anti-mouse/rabbit secondary antibodies for 2 hours at room temperature.

Protein bands were visualized using Immobilon Western Chemilum HRP Substrate (Millipore, USA) on a ChemiDoc MP Chemiluminescent Imager (Bio-rad, USA). Finally, densitometric analysis of band intensity was performed using ImageJ software and the quantitative results were normalized.

**Dual-luciferase reporter assay**

To evaluate miRNA-target interactions, GC cells were seeded into 6-well plates and co-transfected with either the wild-type or mutant 3′-UTR luciferase reporter plasmids along with miR-151a-5p mimics or negative control oligonucleotides using Lipofectamine 3000 (Invitrogen, USA). After 48 h, cells were lysed, and luciferase activities were measured using the Dual-Luciferase Reporter Assay System (Promega, USA) according to the manufacturer’s protocol. Firefly luciferase activity and Renilla luciferase activity were sequentially quantified in each sample after 48 hours using an enzyme labeler. The relative luciferase activity was calculated as the ratio of firefly luciferase activity to Renilla luciferase activity for each transfected well.

**Chromatin immunoprecipitation (****CHIP)-qPCR**

For this experiment, the ChIP kit from Thermo Fisher was used, and the procedure was conducted in accordance with the manufacturer's instructions. To crosslink target proteins and DNA, 37% formaldehyde (final concentration 1%) was added to the cell culture medium and incubated at 37°C for 10 minutes. The crosslinking was terminated by adding glycine (final concentration of 0.125 M) and incubating for 5 minutes at room temperature. Cells were scraped, collected in 15 mL centrifuge tubes, washed twice with pre-cooled PBS, and centrifuged (1000 g, 4°C, 5 min). Detergent-based lysis solution containing protease inhibitors was added, incubated on ice for 10 minutes, centrifuged (5000 g, 5 min, 4°C), and the supernatant discarded. Cells were resuspended in MNase Digestion Buffer, incubated on ice for 10 minutes, sonicated, and centrifuged (10,000 g, 10 min, 4°C) to collect the supernatant. A portion of the sample was analyzed by agarose gel electrophoresis to ensure DNA fragmentation into 200-1000 bp sizes. The remaining samples were diluted with ChIP buffer, mixed with Protein A/G magnetic beads, and incubated at 4°C for 1 hour. Beads were separated using a magnetic rack, and the supernatant retained. Chromatin samples were divided into experimental and control groups, incubated with specific antibodies or isotype IgG overnight at 4°C, washed, and eluted using IP elution buffer. DNA was released by adding NaCl and proteinase K, purified using DNA purification columns, and quantitatively analyzed by qRT-PCR.

**Multiplex immunohistochemistry (mIHC)**

Gastric cancer tissue samples were fixed in 4% paraformaldehyde at room temperature for 24 hours, followed by repeated washes with PBS to remove residual fixative and any precipitates. Tissues were dehydrated through a graded ethanol series, cleared in xylene, and embedded in paraffin. Sections (4–6 μm) were cut, mounted on glass slides, and baked. Deparaffinization was performed in xylene (three times, 5 minutes each), followed by rehydration through descending ethanol concentrations and PBS washing. Antigen retrieval was carried out via microwave heating in retrieval buffer. Non-specific binding sites were blocked with serum to reduce background signal. Primary antibody working solution was applied evenly and incubated overnight under appropriate conditions. After buffer washing, HRP-conjugated secondary antibody was added and incubated at room temperature for 1 hour. Following additional washes, fluorescent dye working solution (diluted 1:100) was applied to fully cover the tissue area, incubated at room temperature for 10 minutes, and then washed again. The staining procedure was repeated for sequential immunolabeling with different antibodies. Finally, sections were mounted and examined under a fluorescence microscope.

**Immunofluorescence (IF)**

Cells were seeded onto confocal culture dishes and cultured until confluence. The medium was removed and cells were washed three times with ice-cold PBS. Samples were sequentially fixed with immunostaining fixative, permeabilized with permeabilization buffer, and blocked with blocking solution, with three 5-minute washes in PBS on a shaker after each step. Primary antibody working solution was added and incubated overnight at 4 °C on a rotator. The following day, cells were washed three times (5 minutes each) with PBS, followed by incubation with fluorescently labeled secondary antibody at room temperature for 90 minutes under light-protected conditions. After secondary antibody staining, nuclei were counterstained with DAPI solution for 15 minutes at room temperature in the dark. Cells were then washed three additional times with PBS (5 minutes each). Following completion of all staining steps, samples were imaged using a laser scanning confocal microscope, with consistent acquisition settings maintained across all samples within the same experimental batch.

**Cytokine Array Analysis**

Cytokine expression in gastric cancer cell supernatants was analyzed using the Human XL Cytokine Array Kit (R&D Systems) following the manufacturer’s protocol. Conditioned media were collected after 48-hour culture, centrifuged, and incubated with the array membrane overnight at 4 °C. After sequential incubations with detection antibodies and Streptavidin-HRP, signals were visualized using chemiluminescence and captured with an automated imaging system.

**Animal experiments**

Female NODPrkdcem26Cd52Il2rgem26Cd22/NjuCrl (NCG) immunodeficient mice were purchased from GemPharmatech LLC. (Nanjing, China) and randomly assigned to different experimental groups. Gastric cancer xenograft models were established by subcutaneous injection of gastric cancer cells into the right axillary region. When tumor volume reached 50–100 mm³, mice were subjected to subsequent treatments. T cells were first activated with anti-CD3/CD28 antibodies and then co-cultured with macrophages or exosome-treated macrophages for 24 hours. These cells were administered to mice via intraperitoneal injection on days 0, 2, and 4. Starting from day 5, mice received intraperitoneal injections of either LAG3 neutralizing antibody or IgG isotype control every five days according to group assignment. On day 30, mice were euthanized and tumors were harvested, photographed, and weighed. Fresh tumor tissues were rapidly processed into single-cell suspensions; portions were snap-frozen in liquid nitrogen, and others were fixed in formaldehyde for downstream analysis. All animal experiments were approved by the Ethics Committee of Laboratory Animal Center, Wuxi School of Medicine, Jiangnan University (JN. No20240630b0801001[389]).

**Statistical analysis**

Statistical analysis were performed using GraphPad Prism 9.0. Data are presented as mean ± standard deviation (SD). For parametric variables, Student’s t-test was used; for non-parametric variables, the chi-square test (χ² test) was applied. Survival analysis was conducted using the Kaplan-Meier method. A p-value < 0.05 was considered statistically significant.
